# Interfacial “Double-Terminal Binding Sites” Catalysts Synergistically Boosting the Electrocatalytic Li_2_S Redox for Durable Lithium–Sulfur Batteries

**DOI:** 10.1021/acsnano.3c11903

**Published:** 2024-03-11

**Authors:** Huifang Xu, Qingbin Jiang, Kwan San Hui, Shuo Wang, Lingwen Liu, Tianyu Chen, Yunshan Zheng, Weng Fai Ip, Duc Anh Dinh, Chenyang Zha, Zhan Lin, Kwun Nam Hui

**Affiliations:** † Joint Key Laboratory of the Ministry of Education, Institute of Applied Physics and Materials Engineering, University of Macau, Avenida da Universidade Taipa, Macau SAR 999078, People’s Republic of China; ‡ Department of Physics and Chemistry, Faculty of Science and Technology, University of Macau, Taipa, Macau SAR 999078, People’s Republic of China; § NTT Hi-Tech Institute, Nguyen Tat Thanh University, Ho Chi Minh City 700000, Vietnam; ∥ School of Engineering, Faculty of Science, University of East Anglia, Norwich NR4 7TJ, United Kingdom; ⊥ School of Chemical Engineering and Light Industry, Guangdong University of Technology, Guangzhou 510006, People’s Republic of China

**Keywords:** separator architecture, double-terminal binding sites, superb electrocatalysis, energy barriers, binding energy

## Abstract

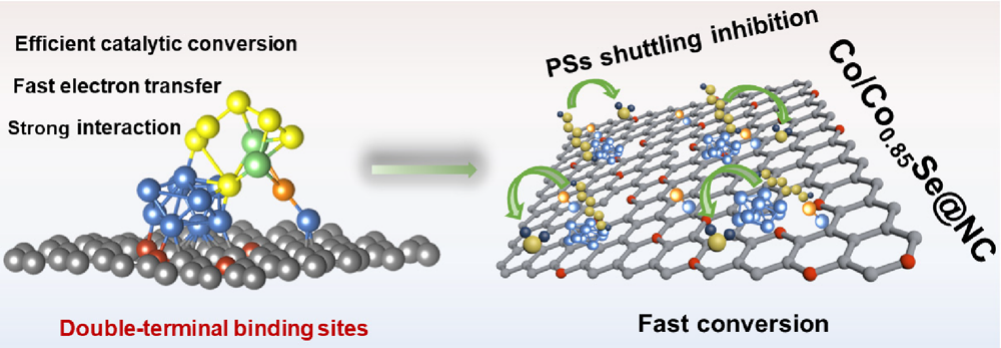

Catalytic conversion of polysulfides emerges as a promising approach to improve the kinetics and mitigate polysulfide shuttling in lithium–sulfur (Li–S) batteries, especially under conditions of high sulfur loading and lean electrolyte. Herein, we present a separator architecture that incorporates double-terminal binding (DTB) sites within a nitrogen-doped carbon framework, consisting of polar Co_0.85_Se and Co clusters (Co/Co_0.85_Se@NC), to enhance the durability of Li–S batteries. The uniformly dispersed clusters of polar Co_0.85_Se and Co offer abundant active sites for lithium polysulfides (LiPSs), enabling efficient LiPS conversion while also serving as anchors through a combination of chemical interactions. Density functional theory calculations, along with *in situ* Raman and X-ray diffraction characterizations, reveal that the DTB effect strengthens the binding energy to polysulfides and lowers the energy barriers of polysulfide redox reactions. Li–S batteries utilizing the Co/Co_0.85_Se@NC-modified separator demonstrate exceptional cycling stability (0.042% per cycle over 1000 cycles at 2 C) and rate capability (849 mAh g^–1^ at 3 C), as well as deliver an impressive areal capacity of 10.0 mAh cm^–2^ even in challenging conditions with a high sulfur loading (10.7 mg cm^–2^) and lean electrolyte environments (5.8 μL mg^–1^). The DTB site strategy offers valuable insights into the development of high-performance Li–S batteries.

## Introduction

Lithium–sulfur (Li–S) batteries are considered among the most promising alternatives to Li-ion batteries owing to their ultrahigh intrinsic energy density (2600 Wh kg^–1^), which far exceeds that of commercial battery systems.^1,2^ In addition to the high theoretical specific capacity of 1675 mAh g^–1^, the sulfur cathode boasts the advantages of natural abundance, environmental friendliness, and low cost.^3^ Nevertheless, commercialization of Li–S batteries is still challenged by uncontrolled dendritic growth of lithium metal anodes,^4^ sluggish sulfur redox reactions,^5^ and unsatisfactory “shuttling” behavior of soluble lithium polysulfide (LiPSs) intermediates.^6^ During cycling, LiPSs dissolve into the organic electrolyte and then migrate from the sulfur cathode to the Li anode, resulting in the loss of active material and the corrosion of lithium metal (Figure S1a).^7−9^ Among these knotty hurdles, the intrinsic large energy barrier caused by the phase transformation of polysulfide to Li_2_S is a critical issue because it not only occupies three-quarters of the total battery theoretical capacity but is also closely related to other challenges,^10^ especially intensifying LiPSs’ shuttling as polysulfide cannot be converted promptly.^11^ Thus, fundamentally improving the reaction kinetics and alleviating the shuttle effect of LiPSs play key roles in achieving the desirable electrochemical performance of Li–S batteries. Furthermore, to maximize the high energy density of Li–S batteries in practical implementations, a lean electrolyte condition and high sulfur loading are required.^12^ Unfortunately, these requirements further aggravate the drawbacks as we mentioned before and increase the electrochemical polarization, leading to low Coulombic efficiency, low sulfur utilization, and rapid capacity degradation.^13,14^

To conquer these issues, various strategies have been proposed to suppress the LiPSs’ shuttling and promote sulfur transformation reactions. Among them, introducing functional sites into the meso-/microporous carbonaceous frameworks has been evaluated as one of the most efficient strategies. Abundant pore sizes can reduce the ion/electron transport distance, provide a larger specific surface area for Li_2_S deposition, and physically constrain LiPSs diffusion,^15^ while functional sites are dedicated to immobilizing LiPSs and increasing catalytic activity (Figure S1b).^16^ These sites are mainly divided into three categories: polar materials and single-atom and metal catalysts with various aggregation extents (clusters, nanoparticles).^17,18^ Polar materials,^19−21^ such as metal oxides,^22,23^ metal phosphides,^24^ metal nitrides,^25,26^ metal borides,^27^ metal carbons,^28−30^ metal sulfides,^31−33^ and metal selenides,^34−36^ mainly rely on the strong chemical interaction between the anchoring sites and polysulfides to immobilize polar LiPSs due to its polar surface properties. In addition, some polar materials with high electrical conductivity show catalytic effects for the sulfur redox reaction, especially transition metal selenides, which are a class of compounds with “metal-like conductivity” properties.^37^ On the other hand, single-atom and metal catalysts,^38,39^ such as Zn single atoms,^40^ Fe single atoms,^41,42^ Co nanoparticles,^43−45^ and Ni nanoparticles,^46^ can catalytically convert LiPS intermediates because of the better conductivity and the immobilization of LiPSs by the binding effect of the efficient charge transfer from the metal center to neighboring molecules driven by the delocalization of unpaired electrons. Moreover, they can facilitate the immobilization of LiPSs by the binding effect between the central metal atom and polysulfides via a Lewis acid–base interaction.^44^ Among them, Co exhibits a lower intrinsic spin state due to its 3d orbital electron configuration t^6^_2g_e^1^_2g_ features.^43,45^ Although progress has been made, the performance of Li–S batteries is still unsatisfactory because most functional sites in previous studies can be considered single-terminal binding (STB) sites. The utilization of the STB sites still presents a significant challenge in achieving synchronous immobilization and conversion of LiPSs and effectively addressing their shuttle effect at the root,which depends on multiple factors such as binding affinity, ion diffusion, number and dispersion of active sites, and charge transfer rate.^37,47−49^ Recently, Zhao et al. designed the double-terminal binding (DTB) site strategy to simultaneously facilitate LiPSs’ conversion over Co single-atom and anchoring LiPSs over a combination of polar ZnS sites and Co single-atom sites, which have received substantial research interest in the field.^50^

Although the realization of atomic-level dispersity and ultrahigh atom utilization of single-atom catalysts is useful for the elevated performance in the catalytic conversion of LiPSs,^18,51^ their concentration in carbon matrices is typically low, usually below 5 wt %. This condition results in a catalyst/S atomic ratio of approximately 1:47 in a typical battery, limiting the amount of active sites and adversely affecting highly efficient LiPS conversion reactions.^52,53^ To achieve high-performance Li–S batteries, especially under high sulfur loading, developing catalysts with DTB sites, such as clusters with abundant interfaces,^48^ that can simultaneously provide both high dispersion and an abundance of active sites is a priority. It is worth noting that prior research efforts have predominantly focused on the design of various heterojunctions based on polar materials due to their demonstrated enhancements in electrocatalytic performance. However, a common issue with these designed heterojunctions is their tendency to accumulate, resulting in an inefficient utilization of catalyst sites. Furthermore, most studies have primarily concentrated on investigating the catalytic activity or adsorption capabilities of sulfur species using single polar materials with STB sites, while explorations into interfaces through DTB sites are in the early stages and lack a systematic approach.^54−56^ In addition, gaining an understanding of the polysulfide conversion mechanism at the atomic scale through advanced *in situ* characterization techniques and density functional theory (DFT) calculations is also of great value.^57,58^

In this work, we present a fundamental investigation into the adsorption and catalytic mechanisms of sulfur species on the interfaces with DTB sites. In detail, dodecahedral mesoporous conductive frameworks containing uniformly *in situ* embedded polar Co_0.85_Se and Co clusters was developed as an effective electrocatalyst, which form abundant interfaces with DTB sites. The combination of DFT calculations and *in situ* Raman and X-ray diffraction (XRD) techniques suggests that the interfaces with DTB sites provide abundant active catalytic sites, increased adsorption of LiPSs, and a lower energy barrier in LiPSs’ convention compared with STB sites, thereby boosting redox reaction kinetics and eliminating lithium metal corrosion and the shuttle effect in Li–S batteries. As a result, the as-prepared Co/Co_0.85_Se@NC-based Li–S batteries exhibit a high and stable discharging capacity (932 mAh g^–1^ after 200 cycles, 0.5 C), ultralong cycling life (1000 cycles with a low-capacity fade rate of 0.042% at high current density of 2 C), excellent rate capability (849 mAh g^–1^ at 3 C), and high Coulombic efficiency. Even at a high sulfur loading of 10.7 mg cm^–2^ and a lower electrolyte/sulfur ratio of only 5.8 μL mg^–1^, the areal capacity of the sulfur cathode can achieve 10.0 mAh cm^–2^, indicating the effectiveness of the rationally designed Co-based compounds. Our strategy of Co/Co_0.85_Se@NC construction offers valuable insight into the design of DTB materials en route toward the desired performance of Li–S batteries.

## Results and Discussion

### Material Characterizations

**Figure 1:**
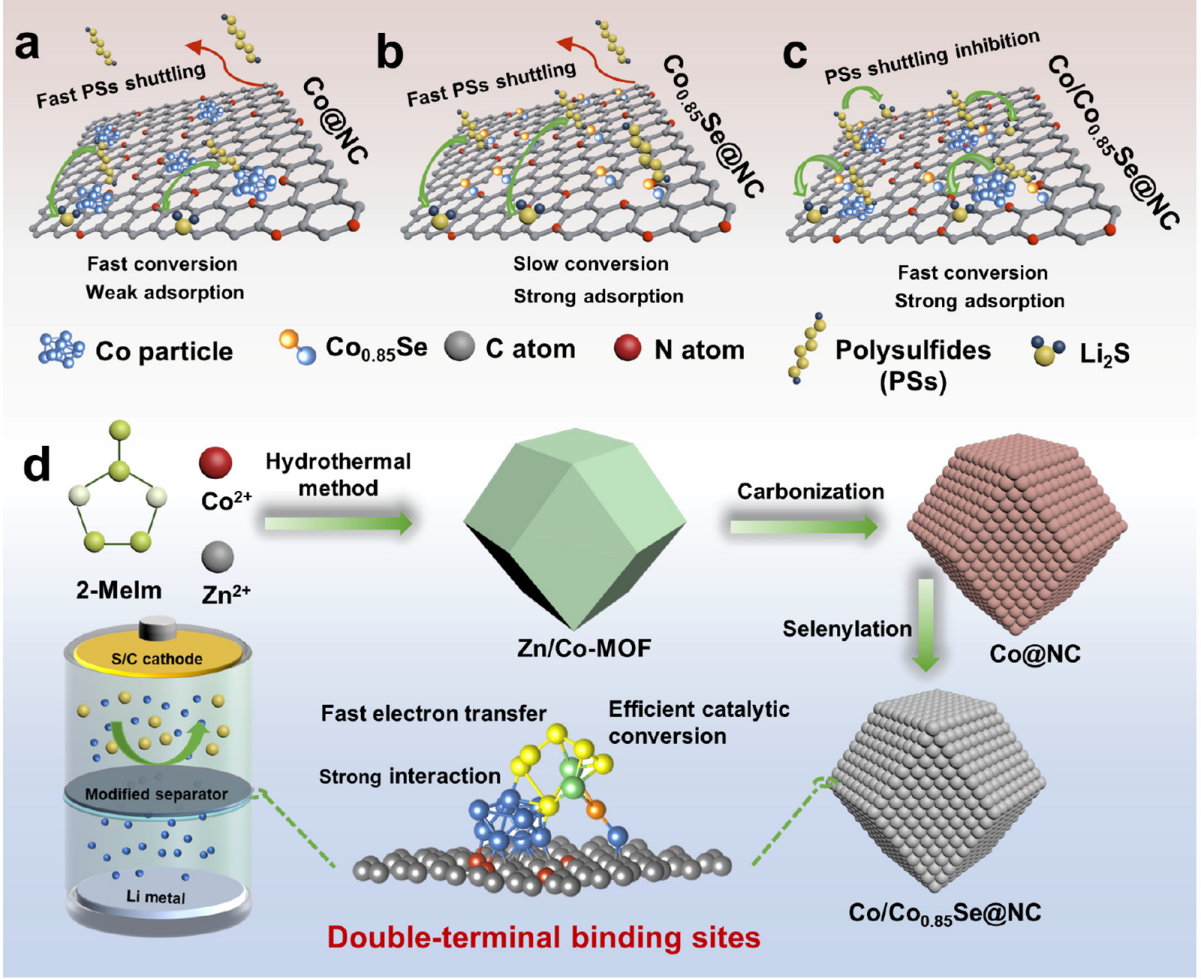
Schematic illustrations of the DTB sites. (a–c) Illustrations of catalyst design. (d) Illustration of the synthesis route of the Co/Co_0.85_Se@NC.

Figure 1a–c illustrates the design rationale of the regular dodecahedral N-doped framework embedded with polar Co_0.85_Se and Co clusters. Co clusters exhibit high dispersion and good catalytic activity due to their superior conductivity. However, the nonpolar surface of Co metal leads to a relatively weak mutual affinity between Co and polysulfides, allowing shuttle behavior to persist. On the other hand, polar Co_0.85_Se demonstrates strong adsorption for LiPSs owing to its polar surface and the formation of Li–Se bonds during cycling. However, its redox activity is comparatively lower than that of the metal catalyst, thus still allowing shuttle behavior. Clearly, the STB sites fail to achieve highly efficient conversion kinetics and complete prevention of LiPS diffusion through the separator to the Li anode over time. As depicted in Figure 1c, the DTB sites, comprising Co clusters and polar Co_0.85_Se sites, possess a unique structure that enables the catalytic oxidation and effective immobilization of soluble LiPS intermediates. This is facilitated by the lower energy barrier in the LiPS convention and the enhanced adsorption of LiPSs (as discussed in the section on DFT). In this type of heterojunction composite, it is commonly observed that the metal component plays a crucial role in facilitating fast electron migration, while the presence of polar materials contributes sufficient binding energy. This observation is consistent with previous studies.^59−62^

Figure 1d illustrates the synthesis route of the regular dodecahedral N-doped framework embedded with polar Co_0.85_Se and Co clusters, which were synthesized by a facial method based on nanostructured ZIF materials. In the first step, the Zn/Co-MOF was constructed through a self-assembly process using cobalt nitrate, zine nitrate, and 2-methylimidazole. The molar ratio of Co and Zn precursors was designed to be 1:2, which ensures that the Co clusters can be uniformly dispersed and provides sufficient Co content to enable abundant active sites for Li–S batteries. Following carbonization treatment, a homogeneous distribution of Co clusters within a nitrogen-doped carbon compound was obtained. Subsequently, the Co/Co_0.85_Se@NC heterostructure was obtained by selenizing the as-prepared Co@NC precursor to Se powder in a ratio of 1:2, whereas Co_0.85_Se@NC was obtained by the same method but with excessive Se powder. The resulting Co@NC, Co_0.85_Se@NC, and Co/Co_0.85_Se@NC samples were coated onto a commercial separator to prepare a multifunctional separator by using a simple blade-coating method.

**Figure 2:**
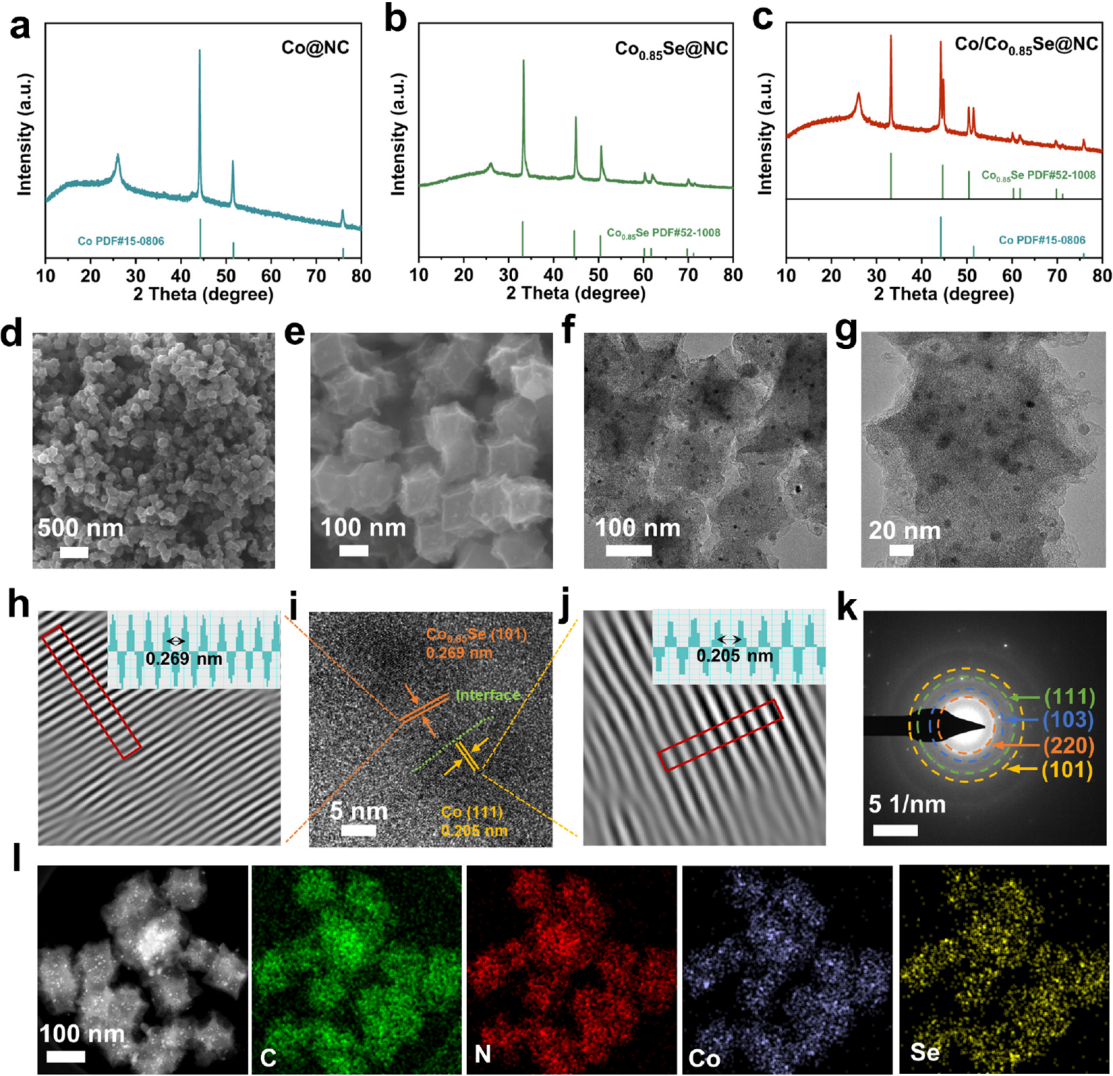
Characterizations of the DTB sites. XRD patterns of (a) Co@NC, (b) Co_0.85_Se@NC, and (c) Co/Co_0.85_Se@NC. (d, e) SEM images and (f, g) TEM images of Co/Co_0.85_Se@NC. (i) High-resolution TEM image and (h, j) the IFFT lattice images of the selected area of Co/Co_0.85_Se@NC (inset: lattice distance profiles of the area in red). (k) SAED pattern of Co/Co_0.85_Se@NC. (l) TEM-EDX element mapping of Co/Co_0.85_Se@NC.

The Co/Co_0.85_Se@NC DTB sites are obtained by a simple carbonation–selenylation method. The crystal structure of those samples was analyzed by powder XRD patterns (Figures 2a–c and Figure S2). In those product spectra, the peak at 26.2° corresponds to the characteristic carbon peak that originated from the pyrolysis of organic ligands. In addition, the Co@NC product exhibits a series of peaks at 44.3, 52.3, and 76.4° corresponding to the Co (111), (200), and (220) planes, respectively (PDF No. 15-0806). After the selenization process, the characteristic peaks of the Co_0.85_Se@NC product can be observed at 33.2, 44.7, 50.5, 60.3, 61.8, 69.9, and 71.3° corresponding to the (101), (102), (110), (103), (112), and (202) planes of Co_0.85_Se, respectively (PDF No. 52-1008). In addition, in the Co/Co_0.85_Se@NC spectrum, all of the diffraction peaks can be attributed to Co and Co_0.85_Se and verify the presence of those phases in the Co/Co_0.85_Se@NC structure. Moreover, in all spectra, no other evident characteristic peaks are found, indicating no impurity in those products. A scanning electron microscope (SEM) test was applied to observe the morphology of the obtained products. The SEM images of Zn/Co-MOF (Figure S3) show the uniform, typical dodecahedral morphology with a smooth surface and an average particle size of approximately 150 nm. After the carbonation and selenylation process, the morphology of Co@NC, Co_0.85_Se@NC, and Co/Co_0.85_Se@NC shows a structure similar to that of the precursor, indicating that the annealing process did not destroy the morphology of the products (Figure 2d,e and Figures S4 and S5). The transmission electron microscope (TEM) images show that many polar Co_0.85_Se and Co clusters with an average size of 10 nm are homogeneously dispersed in the regular dodecahedral conductive framework (Figure 2f,g). The high-resolution TEM (HRTEM) image in Figure 2i and two inverse fast-Fourier-transform lattice images (Figure 2h,j) of Co/Co_0.85_Se@NC show two distinct fringes with spacings of 0.205 and 0.269 nm, which are in accordance with the (111) facet of Co particles and (101) plane of Co_0.85_Se crystals, respectively. Meanwhile, a selected-area electron diffraction pattern confirmed the formation of Co and Co_0.85_Se DTB sites (Figure 2k). The elemental mapping analysis of C, N, Co, and Se clearly demonstrates that the elements are uniformly distributed in the frameworks of Co/Co_0.85_Se@NC composites, consistent with our rational design (Figure 2l and Figure S6). The elemental mapping analysis of Co@NC and Co_0.85_Se@NC in Figures S7 and S8 also indicates uniform distribution in the frameworks.

**Figure 3:**
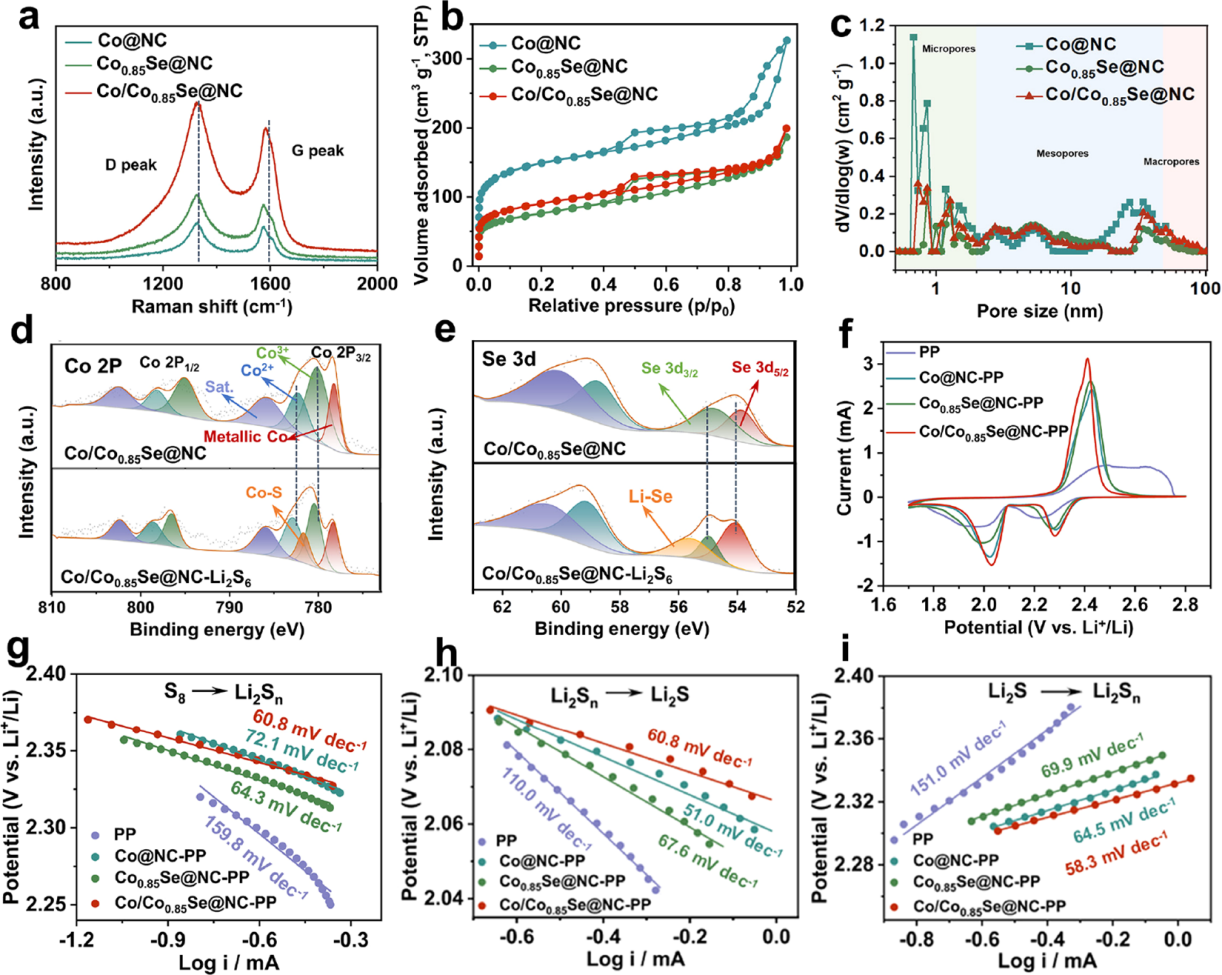
(a) Raman spectra, (b) nitrogen adsorption desorption isotherms, and (c) pore size distributions of Co@NC, Co_0.85_Se@NC, and Co/Co_0.85_Se@NC. High-resolution XPS spectra of (d) Co 2p and (e) Se 3d of Co/Co_0.85_Se@NC before and after adsorption of Li_2_S_6_. (f) CV profiles of various separators at a scan rate of 0.1 mV s^–1^ and corresponding Tafel plots of (g) S_8_→ Li_2_S*_n_*, (h) Li_2_S_*n*_ → Li_2_S, and (i) Li_2_S → Li_2_S_*n*_.

Figure 3a shows the Raman spectra of Co@NC, Co_0.85_Se@NC, and Co/Co_0.85_Se@NC. All products exhibit strong Raman peaks at 1325 and 1596 cm^–1^, which are attributed to the D band (sp^3^ defects of disordered carbon) and G band (the in-plane vibration of graphitic layers) of the carbon. The high *I*_D_/*I*_G_ values of Co@NC (1.13), Co_0.85_Se@NC (1.17), and Co/Co_0.85_Se@NC (1.16) suggest numerous defects in NC caused by the calcining process. The Brunauer–Emmett–Teller specific surface area of the Zn/Co-MOF precursor is up to 1335 m^2^ g^–1^ (Figure S9). Despite the calcination process, the obtained frameworks still preserve a large pore volume and a high specific surface area, as shown in Figure 3b and Table S1, owing to the precursor and appropriate heating conditions under a low heating rate of 2 °C min^–1^ to maintain the structure of the Zn/Co-MOF precursor. The pore size distribution of Co/Co_0.85_Se-NC shown in Figure 3c exhibits abundant micro-, meso-, and macropores. The large specific surface area and diverse pore structure are favorable for fast Li-ion diffusion and sustainable adsorption of soluble LiPS molecules and electrolytes.

X-ray photoelectron spectroscopy (XPS) was used to verify the chemical environments of the three catalysts and the newly formed bonding structures after absorbing Li_2_S_6_. The full-scan survey spectrum of these samples and the high-resolution elemental spectrum indicate the presence of C, N, Se, and Co (Figures S10–S12). The C 1s spectrum exhibits distinctive peaks at 284.8 and 285.9 eV, corresponding to C=C and C=N, respectively. In the N 1s spectrum, the peaks at 398.5, 400.3, and 400.9 eV are assigned to pyridinic, pyrrolic, and graphitic N species, respectively. Compared with the binding energies of Co 2p in Co@NC (778.2 eV for metal Co and 780.1 eV for Co–N) and Co_0.85_Se@NC (780.2 eV for 2p_3/2_ and 782.3 eV for 2p_5/2_), the shifted binding energy of Co 2p in Co/Co_0.85_Se-NC (778.2 eV for metal Co, 780.2 eV for 2p_3/2_, and 782.3 eV for 2p_5/2_) suggests the formation of the Co/Co_0.85_Se-NC composite. Figures S11e and S12e show two characteristic peaks located at 53.8 and 54.8 eV in the fitted high-resolution Se 3d spectrum and two distinct peaks in the range of 52–57 eV, which can be attributed to 3d_3/2_ and 3d_5/2_. XPS data on the Co@NC, Co_0.85_Se@NC, and Co/Co_0.85_Se@NC powder after absorbing Li_2_S_6_ were collected. The presence of the Co–S bond at 781.7 eV in the Co 2p spectra of Co@NC (Figure S13a) and Co/Co_0.85_Se@NC samples (Figure 3d) and the higher binding energy of Co^3+^ (780.6 eV) and Co^2+^ (782.8 eV) in Co_0.85_Se@NC (Figure S13b) and Co/Co_0.85_Se@NC are further indications of the successful chemisorption of Li_2_S_6_ on the catalytic surface. Moreover, after trapping Li_2_S_6_, Se 3d peaks of Co_0.85_Se@NC-Li_2_S_6_ and Co/Co_0.85_Se@NC-Li_2_S_6_ display a distinct shift toward higher binding energy, with values of 0.3 and 0.2 eV, accompanied by a new peak at 55.5 eV, indicating the formation ofa Li–Se bond (Figure 3e and Figure S13c).

### Electrochemical Performance

**Figure 4:**
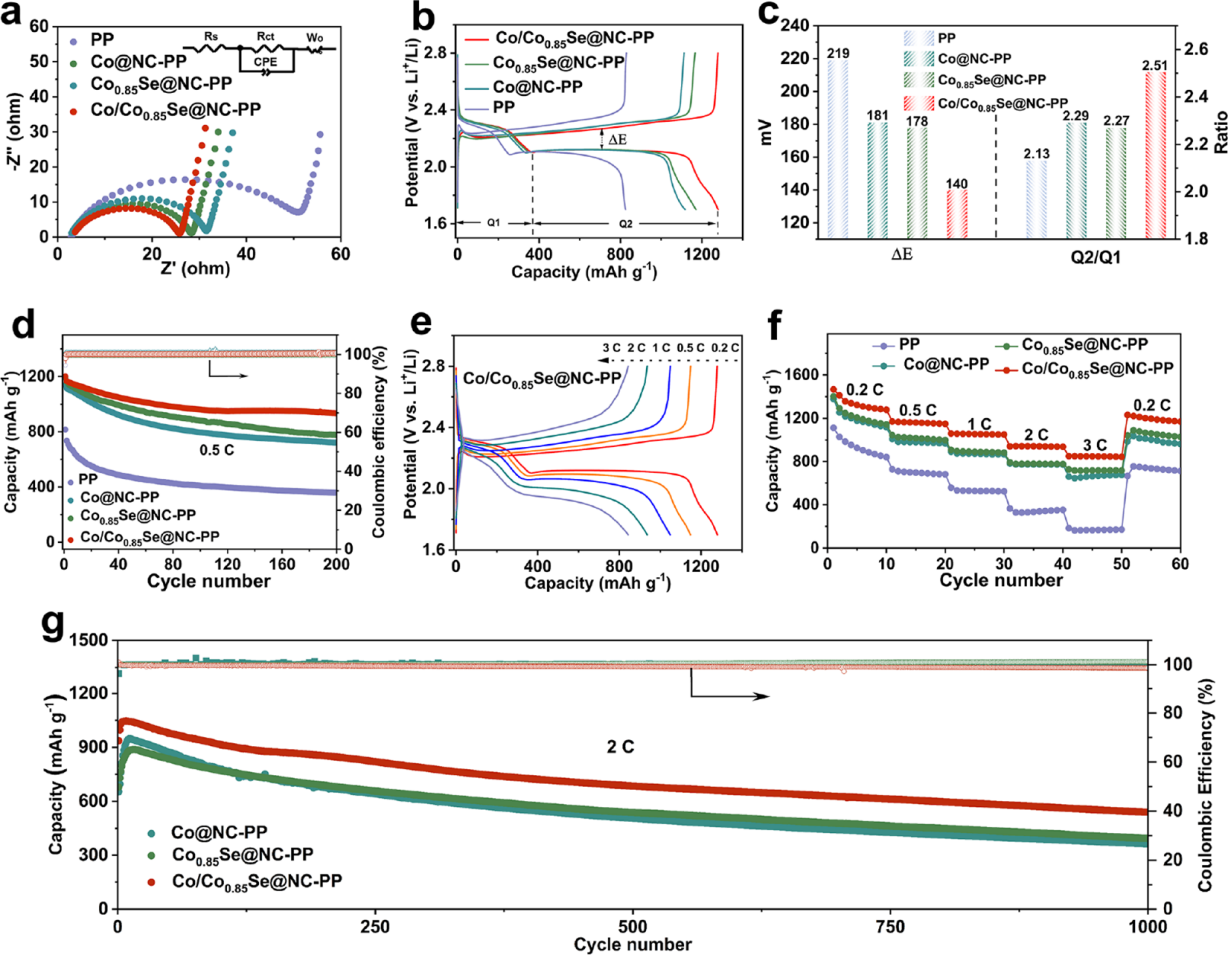
Electrochemical properties of Li–S cells. (a) EIS spectra, (b) charging and discharging curves with a 0.2 C current rate, (c) Δ*E* and Q2/Q1 values obtained at 0.2 C, (d) cycling performance at 0.5 C, (e) galvanostatic charge/discharge profiles under different rates, (f) rate performance under different rates, and (g) long-term cycle performance at 2 C of Li–S cells with Co@NC-modified, Co_0.85_Se@NC-modified, and Co/Co_0.85_Se@NC-modified separators.

Electrochemical performance is considered to systematically evaluate the catalytic ability of Co@NC, Co_0.85_Se@NC, and Co/Co_0.85_Se@NC catalysts in Li–S batteries. Sulfur was infiltrated in commercial Super-P by a typical melt diffusion method, resulting in a C/S composite with 70 wt % sulfur content as determined by TGA (Figure S14). All the cells were assembled by a C/S composite as the cathode and Li foil as the counter electrode, and the different modified commercial PP materials acted as the separator. The coating layers (Figures S15 and S16), which were 7 μm thick, strongly adhered to the PP substrate and remained stable without mechanical issues when subjected to bending and wrinkling. SEM was utilized to study the morphologies of various modified separators. As illustrated in Figure S17, the modification layers display a relatively smooth surface, indicating uniform adhesion of the coating layer onto the blank PP surface. This suggests the formation of a dense protective layer without cracks, effectively preventing the shuttle of polysulfides through the separator and their participation in a negative electrode reaction. The polarity of materials used for modifying separators plays a crucial role in facilitating the adsorption of polysulfides and uptake of the electrolyte. In the contact angle tests, the contact angle between the DME/DOL electrolyte and Co/Co_0.85_Se@NC-PP was measured to be 6°, which is lower compared to PP (33°), Co@NC-PP (18°), and Co_0.85_Se@NC-PP (14°) (Figure S18). The results indicate that Co/Co_0.85_Se@NC-PP exhibits a higher level of polarity, allowing for better wettability by the DME/DOL electrolyte. The polar nature of the Co/Co_0.85_Se@NC surface enhances the utilization of polysulfides, thereby contributing to an improvement in the electrochemical performance. Figures 3f and Figure S19 display the cyclic voltammetry (CV) curves of the cells with blank-PP and three modified separators in a voltage window of 1.7–2.8 V. It is evident that both reduction peaks are located at 2.27 and 2.04 V, corresponding to the reduction reaction from sulfur to long-chain LiPSs and further reduction to Li_2_S_2_/Li_2_S. In addition, the oxidation peak at 2.27–2.45 V indicates the oxidation reaction from Li_2_S to sulfur. Moreover, in the three initial cycles, the CV curves of the cell are almost overlapping, indicating the excellent reversibility of the redox reaction in the cell. The results of the CV curves of the three catalysts suggest that the cells with the modified separator display a significantly higher electrocatalytic performance than the blank separator, as evidenced by the sharper redox peaks and smaller overpotential. Further analysis of the Tafel slopes during the oxidation and reduction processes show that the Co/Co_0.85_Se@NC DTB sites exhibits the smallest slope values, indicating more efficient charge transfer for catalyzing the LiPSs conversion and oxidating the discharge product (Figure 3g–i). These results suggest that the use of the DTB structure with bidirectional catalytic activity can significantly improve the electrochemical performance of Li–S batteries. The electrochemical impedance spectroscopy (EIS) spectrum of the four types of cells is shown in Figure 4a. Compared with the cell with a blank separator, that with a modified separator delivers a smaller charge transfer resistance (*R*_ct_) in the high-frequency region of Nyquist plots, showing higher conductivity and better electrochemical kinetics.

Galvanostatic charge/discharge profiles were collected at a current of 0.2 C, as shown in Figure 4b. The discharging and charging profiles of the four cells exhibit two plateaus (reduction process: S_8_ → Li_2_S_8_ → Li_2_S_6_ →Li_2_S_4_ and Li_2_S_4_ → Li_2_S_2_ →Li_2_S) and a plateau (oxidation process: Li_2_S → S_8_), consistent with the CV results. The potential difference (Δ*E*) between the oxidation and reduction plateaus at 50% discharge capacity is indicative of the polarization associated with the redox reaction. We found that the Co/Co_0.85_Se@NC DTB sites exhibit the lowest polarization potential of approximately 140 mV, which was significantly lower than those of both STB sites (approximately 180 mV). This finding suggests that Co/Co_0.85_Se@NC has a synergistic effect that enhances the redox reaction kinetics of LiPSs. Furthermore, the catalytic activity of the catalysts was investigated by comparing the Q2/Q1 ratio (Figure 4c). A heightened Q2/Q1 ratio signifies successful conversion to Li_2_S, thereby denoting a higher catalytic activity of the catalyst. The Co/Co_0.85_Se@NC DTB sites show the highest Q2/Q1 value of 2.51, which is much higher than the Q2/Q1 values of both STB sites. This excellent catalytic activity of DTB sites is attributed to the effective synergistic effect between Co@NC and Co_0.85_Se@NC in accelerating the conversion of LiPSs.

As shown in Figure 4d, the cycling performance of the cells with the blank and Co@NC-, Co_0.85_Se@NC-, and Co/Co_0.85_Se@NC-modified separators was investigated at 0.5 C. The three modified separators have initial discharge capacities of 1201, 1189, and 1138 mAh g^–1^ higher than those of the cell with a blank separator, respectively. More importantly, the Co/Co_0.85_Se@NC-based cell delivers a stable reversible capacity of 932 mAh g^–1^ after 200 cycles with a low capacity decay of 0.11% per cycle, which is more stable than the other cells with the Co_0.85_Se@NC (0.17%)- and Co@NC-modified (0.18%) separators and blank separator (0.28%).

The ultrastable long-cycle electrochemical performance of the Co/Co_0.85_Se@NC-based cell reveals that the DTB sites can greatly improve the utilization of active substances. The rate performance of these cells was investigated in different current densities from 0.2 to 3 C between 1.7 and 2.8 V. The charge and discharge profiles of Li–S cells with modified separators are maintained with different current rates even high rates, which demonstrate the enhanced performance compared with a blank separator (Figure 4e and Figure S20). As shown in Figure 4f, the cell with the Co/Co_0.85_Se@NC-modified separator shows the highest specific capacity of 1466 mAh g^–1^ at 0.2 C, 1170 mAh g^–1^ at 0.5 C, 1057 mAh g^–1^ at 1 C, 940 mAh g^–1^ at 2 C, and 849 mAh g^–1^ at 3 C. When the current rate is returned to 0.2 C, a durable capacity of 1230 mAh g^–1^ can be recovered, which indicates desirable electrochemical performance, structural robustness, and good stability of the DTB sites. The well-defined and reversible dual plateaus were delivered in varying current density conditions even at 3 C, corresponding to the superior rate performance. In addition, the long-term cycles were investigated to further evaluate the outstanding electrochemical performance of the Co/Co_0.85_Se@NC-based cell at a high current density, and the result is shown in Figure 4g. As such, the cell shows outstanding cycling stability with a higher discharge capacity of 539 mAh g^–1^ even after 1000 cycles at 2 C with an ultralow fading rate of 0.042% per cycle. This finding further verified the rational design of the Co/Co_0.85_Se@NC DTB sites in the Li–S batteries. The morphology and crystal structure of Co/Co_0.85_Se after cycling were investigated by using SEM and TEM tests and digital images. The battery was disassembled to showcase the durability of the modification layer after cycling. The modified membrane layers exhibit a surface morphology similar to that observed before cycling, indicating a strong adhesion of the catalysts to the PP substrate and their stability (Figures S21 and S22). Furthermore, TEM characterization of Co/Co_0.85_Se@NC particles after cycling reveals that the Co/Co_0.85_Se @NC component remained unchanged throughout the sulfur redox process (Figure S23). In terms of enhanced capacity, outstanding rate performance, long cycle life, and high Coulombic efficiency, such a separator configuration that contains *in situ* embedded uniformly polar Co_0.85_Se and Co clusters catalytically can boost redox reaction kinetics and eliminate lithium metal corrosion and the shuttle effect, thereby systematically improving the electrochemical performance of robust Li–S batteries.

### Mechanism Analysis

**Figure 5:**
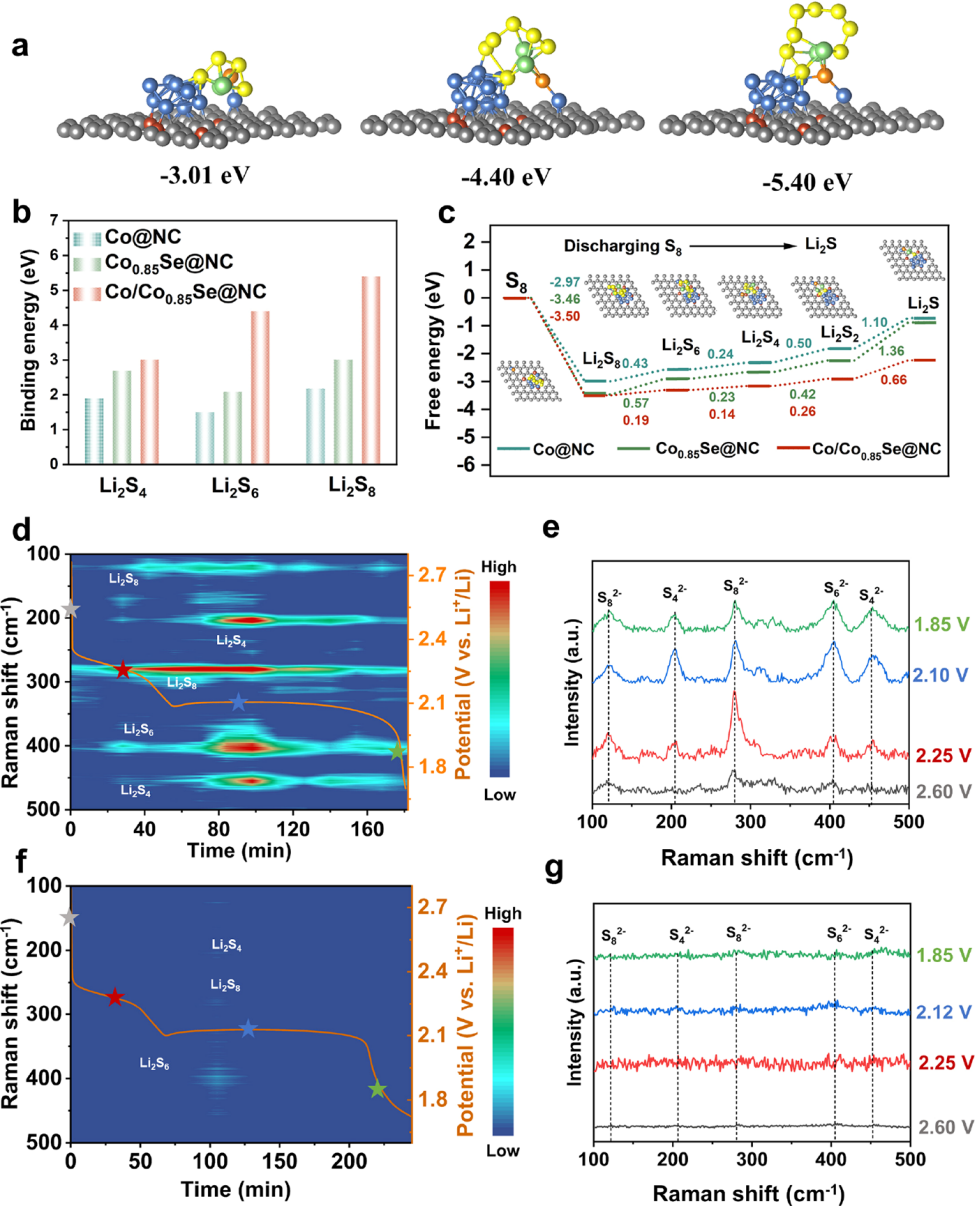
DFT calculations and *in situ* techniques on polysulfide adsorption and redox reactions. (a) Optimized configurations of Li_2_S_8_, Li_2_S_6_, and Li_2_S_4_ on the Co/Co_0.85_Se@NC surface. (b) Adsorption energy between polysulfide and catalysts and (c) energy profiles for the reduction from S_8_ to Li_2_S on Co@NC, Co_0.85_Se@NC, and Co/Co_0.85_Se@NC surfaces. *In situ* Raman of Li–S cells using a (d, e) blank separator and (f, g) Co/Co_0.85_Se@NC-modified separator.

To investigate the mechanism of LiPS capture and conversion by different components, DFT calculations were performed to verify the adsorption and migration of LiPSs on STB and DTB sites. Figure 5a and Figures S24–S26 show the optimized configurations of polar Co_0.85_Se@NC, Co@NC, and Co/Co_0.85_Se@NC and the most stable LiPSs on the surface of the three catalysts. It is worth noting that the Li^+^ bonds with the Se atom in the case of polar Co_0.85_Se@NC and S^2–^ bonds with Co atoms in the case of Co@NC clusters, while an Li–Se bond and S–Co bond form on the Co/Co_0.85_Se@NC surface. The obtained XPS spectra after a Li_2_S_6_ adsorption test demonstrate that the Li atoms tend to bond with Se, while the Co clusters on the surface of carbon are favorable binding sites for S in Li_2_S_6_, correlating well with the Li–Se and Co–S peaks. The adsorption binding energies of Li_2_S_6_ on the surfaces of Co@NC, Co_0.85_Se@NC, and Co/Co_0.85_Se@NC are 1.50, 2.08, and 4.40 eV, respectively, indicating that the DTB sites have the strongest anchoring ability for LiPSs compared with the STB sites (Figure 5b) due to the synergistic effect of polar Co_0.85_Se and Co cluster catalysts. The improved binding energy can effectively immobilize LiPSs and prevent them from shuttling to the anode. In addition, we found that STB and DTB sites have different catalytic properties. We postulate a six-step sulfur conversion reaction (SCR, S_8_ ⇌ Li_2_S) via the S_8_*, Li_2_S_8_*, Li_2_S_6_*, Li_2_S_4_*, Li_2_S_2_*, and Li_2_S*, as shown in Figure S27. In all ideal model systems, single-end sites are adsorption sites, while sulfur-related species are located between double-end sites. The relative energies of all steps in the energy diagrams of the three configurations are at their respective minimum energies. As shown in Figure 5c, the second step S_8_* → Li_2_S_8_* is strongly exothermic in all studied cases, suggesting a strong binding between Li and catalytic sites, resulting in a low energy barrier. The highest energy barrier in the S_8_ → Li_2_S reaction, which is determined by the rate-determining step, can be used as an important parameter for evaluating the SCR activity. For the entire SCR process, the deposition of Li_2_S is the rate-determining step of the complete reaction process, which has the highest Gibbs energy barriers. The calculations indicate that the DTB structure is a relatively superior catalyst to facilitate the conversion of LiPSs and accelerate the formation of Li_2_S, as it has a lower free energy variation (0.66 eV) than Co@NC (1.10 eV) and Co_0.85_Se@NC (1.36 eV).

To discover how the DTB sites improve the performance of Li–S batteries, the working mechanism should be fully understood. Thus, a series of experiments was tested to verify the outstanding anchoring-conversion of LiPSs on Co/Co_0.85_Se@NC. The adsorption ability of Co@NC, Co_0.85_Se@NC, and Co/Co_0.85_Se@NC toward the Li_2_S_6_ solution was evaluated (Figure S28). The color change of the colorful Li_2_S_6_ solution after adding Co@NC, Co_0.85_Se@NC, and Co/Co_0.85_Se@NC powders within 10 min shows that the three catalysts have an adsorption capacity for polysulfide, consistent with the analysis results of XPS. By contrast, the colors of the Li_2_S_6_ solution with Co_0.85_Se@NC and Co/Co_0.85_Se@NC compounds are clearer than that of the solution with Co@NC, indicating that the polar Co/Co_0.85_Se@NC and Co_0.85_Se@NC possess strong adsorption abilities for LiPSs. To assess the effectiveness of the modified separator in suppressing polysulfide diffusion in Li–S batteries, a visual experiment was conducted. Figure S29 shows the left glass chambers filled with polysulfides (Li_2_S_6_) in the DOL/DME solution, while the right chambers contained a colorless blank DOL/DME solution. The two chambers were separated by the commercial separator (top row) or the Co/Co_0.85_Se@NC-modified separator (bottom row). Photographs were taken at 0, 1, 6, and 24 h to observe the change in polysulfide diffusion in different separators. In the top row with the pristine separator, the solution gradually changed from colorless to yellow and eventually turned brown in the right compartment of the H-type glass cell, indicating polysulfide diffusion. UV–vis absorption spectra further confirmed the relationship between the polysulfide concentration in the right compartment and elapsed time. In contrast, the bottom row with the Co/Co_0.85_Se@NC-modified separator exhibited a slightly yellowish color and minimal change in the UV–vis absorption peak after 24 h, indicating effective suppression of polysulfide diffusion by the Co/Co_0.85_Se@NC-modified separator. Figure S30 shows an experimental method of blocking the diffusion caused by the concentration difference in the Li–S battery system. Specifically, the setup condition was to cycle 20 times at 0.2 C, halting at 2.1 V during the 21st discharge sequence, and undergoing a 1 week standstill because of the high content of soluble polysulfides at 2.1 V. During quiescence, the polysulfides would diffuse to the anode where they are reduced to Li_2_S/Li_2_S_2_ on the lithium metal side. Subsequently, the cell undergoes capacity decay due to self-discharge when discharge is continued after 7 days of rest. The irreversible capacity decay of the Co/Co_0.85_Se@NC-based cell is 49 mAh g^–1^, which is much lower than that of the cell using the blank separator (137.2 mAh g^–1^). The results show that the cell with the Co/Co_0.85_Se@NC separator can significantly reduce self-discharge behavior by reducing polysulfide shuttling.

*In situ* Raman spectroscopy was used to evaluate the inhibition of the shuttle effect, and LiPSs signals on the anodic side of the separator were recorded during the discharging process of Li–S batteries (Figure 5d–g). Notably, the Li–S cell with a blank separator exhibits two distinct signals (121 and 279 cm^–1^) from the bending and stretching vibrations of S_8_^2–^ at the beginning of discharge (>2.35 V), indicating the severe shuttle effect of the open-looped S_8_^2–^. In addition, a prominent peak of S_6_^2–^ (near 405 cm^–1^) was observed, mainly attributed to the self-discharged Li–S cell. After the first discharge platform, the signal peak of S_8_^2–^ decreased, and the S_6_^2–^ and S_4_^2–^ signal (202 and 450 cm^–1^) intensities increased due to the reduction of S_8_^2–^. The peak intensities of S_6_^2–^ and S_4_^2–^ reached their maximum as discharging continued. Subsequently, S_6_^2–^ and S_4_^2–^ were further reduced into Li_2_S_2_ and Li_2_S, accompanied by the gradual decrease of their peak intensities. By contrast, weakened LiPSs signals were observed throughout the discharging processes of the Li–S cell with the Co/Co_0.85_Se@NC-modified separator, suggesting the DTB sites’ effectiveness in capturing LiPSs and inhibiting the shuttle effect.

**Figure 6:**
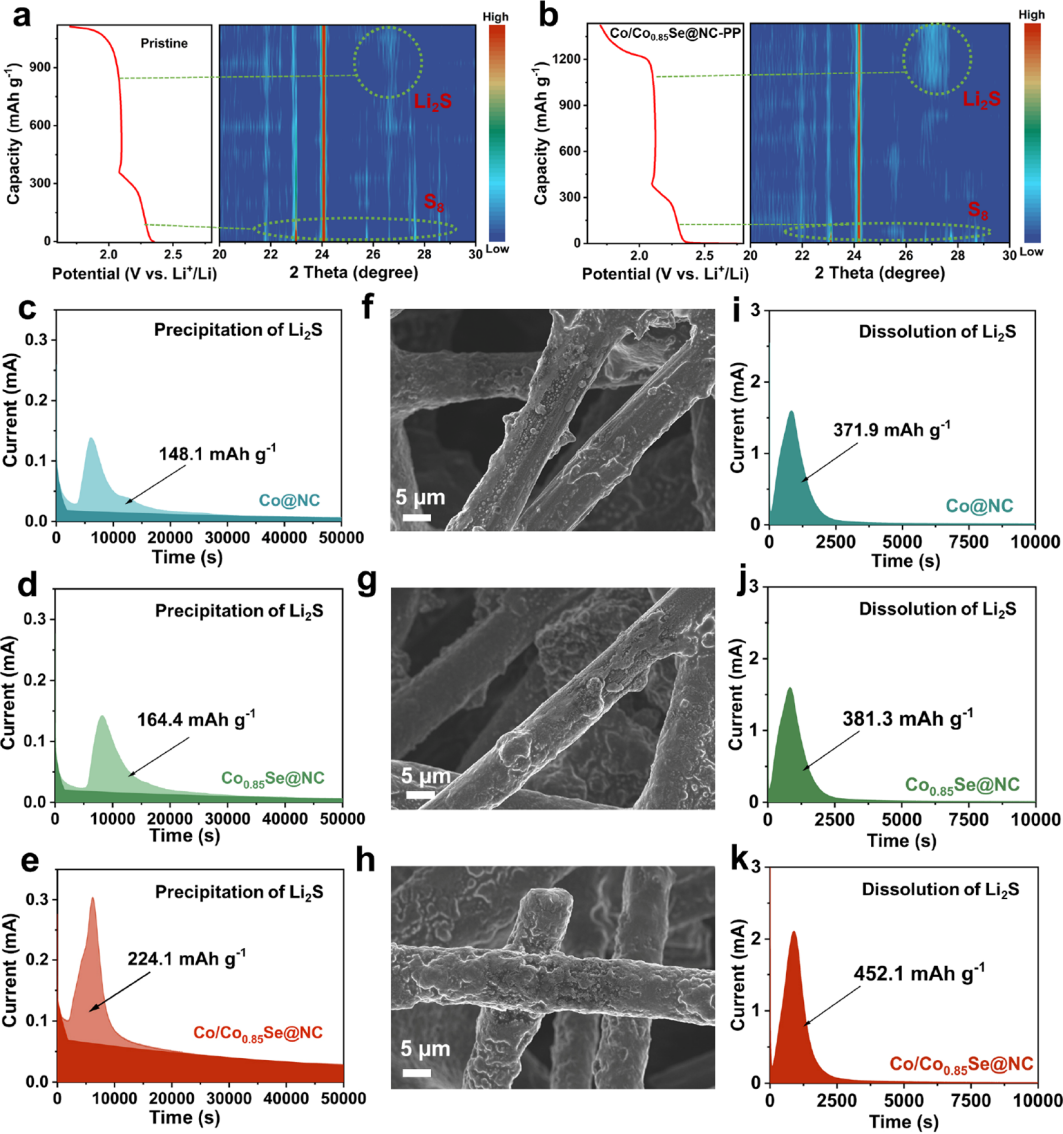
Evaluation of electrocatalytic activity between various electrocatalysts. *In situ* XRD of Li–S cells using a (a) blank separator and (b) Co/Co_0.85_Se@NC-modified separator. (c–e) Li_2_S nucleation test, (f–h) corresponding SEM images after nucleation, and (i–k) Li_2_S dissolution test results on Co@NC, Co_0.85_Se@NC, and Co/Co_0.85_Se@NC surfaces.

*In situ* XRD analysis was conducted on Li–S cells to gain insight into the evolution mechanism of the sulfur species and redox kinetics during the reaction. During the discharging process, S_8_ was reduced to soluble LiPSs, followed by the formation of Li_2_S nanocrystals. Results in Figure 6a,b indicate that at the initial stage of discharge, the diffraction peaks positioned at 23.14, 25.92, 26.82, and 27.8° corresponded to crystalline α-S_8_ (JCPDS No. 008-0247). Consequently, the α-S_8_ peaks disappeared gradually, and a broad diffraction peak at approximately 27.1° emerged, indicating the presence of a cubic Li_2_S phase (JCPDS No. 023-0369), which grew in intensity at the end of discharge. Figure 6a shows a weak Li_2_S peak in the cell with a blank separator. By contrast, a larger area with a higher intensity of the Li_2_S peak is observed in the Co/Co_0.85_Se@NC-based cell, indicating an improved growth and nucleation of the discharge product. These *in situ* experiment results indicate that Co/Co_0.85_Se@NC can not only inhibit the shuttle effect of LiPSs but also effectively regulate LiPSs conversion, thereby improving the electrochemical properties of Li–S batteries.

Electrocatalytic activity is pivotal in facilitating the conversion of LiPSs in addition to the adsorption ability of the active sites. The symmetrical cells were assembled with Co@NC, Co_0.85_Se@NC, and Co/Co_0.85_Se@NC electrodes and Li_2_S_6_-containing electrolyte and then measured with a voltage window within −1 to 1 V at a scan rate of 5 mV s^–1^ to explore the electrocatalysis function of those materials. As shown in Figure S31, the symmetrical cell with Co/Co_0.85_Se@NC displays a higher current density and more evident redox peaks than the two other symmetrical cells, indicating the excellent electrochemical reversibility and better facial polysulfide conversion. The galvanostatic charge–discharge profiles were further investigated to explore the electrocatalytic activity of those compounds. In addition, the kinetics of the Li_2_S nucleation tests were carried out to demonstrate the catalytic performance of Co@NC, Co_0.85_Se@NC, and Co/Co_0.85_Se@NC catalysts. As shown in Figure 6c–e, the potentiostatic discharge curve of Li_2_S deposition from LiPSs at 2.05 V were collected using commercial carbon fiber paper (CP) loaded with Co@NC, Co_0.85_Se@NC, and Co/Co_0.85_Se@NC as the cathodes. The capacity of nuclear Li_2_S conversion was calculated according to the quantity of electric charge based on Faraday’s law (Figure S32). CP-Co/Co_0.85_Se@NC shows a much higher dissolution current response, earlier dissolution time, and highest capacity of Li_2_S precipitation (224.1 mAh g^–1^) compared with those on CP-Co_0.85_Se@NC (164.4 mAh g^–1^) and CP-Co@NC (148.1 mAh g^–1^), which demonstrates the outstanding conversion kinetics of Co/Co_0.85_Se@NC toward the fast conversion of Li_2_S. SEM was utilized to examine the morphologies of Li_2_S deposited on various catalyst supports. A uniform mass of Li_2_S deposition was observed on CP-Co_0.85_Se@NC, whereas Li_2_S aggregation occurred on a part of the CP-Co@NC and CP-Co_0.85_Se@NC surfaces (Figures 6f–h). In the Li_2_S dissolution process, CP-Co/Co_0.85_Se@NC also displayed a much higher dissolution current response and larger dissolution capacity than those of CP-Co@NC and CP-Co_0.85_Se@NC (Figure 6i–k). These findings are consistent with the calculation and experimental results of *in situ* XRD, indicating that the DTB sites boost sulfur conversion reactions.

### Application Studies

**Figure 7:**
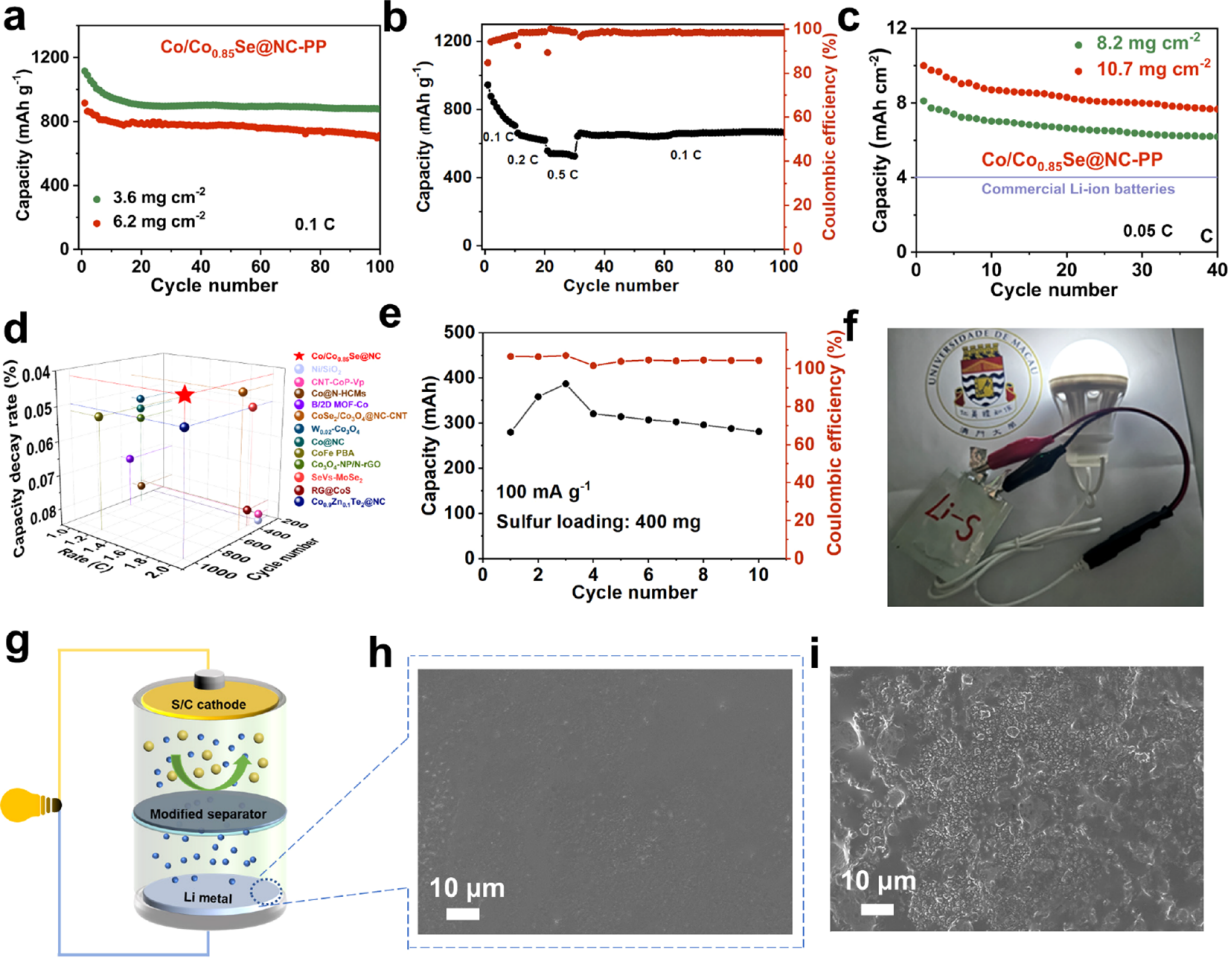
Cycling stability of Li–S cells with high sulfur loading and their practical application. (a) Cycle performance with sulfur loadings of 3.6 and 6.2 mg cm^–2^, (b) rate performance with high sulfur loading of 5.2 mg cm^–2^, and (c) cycling performance with ultrahigh sulfur loadings of 8.2 and 10.7 mg cm^–2^ of cells with a Co/Co_0.85_Se@NC-modified separator. (d) Cell performance comparison of recently reported work in Li–S cells. (e) Cycling performance of 400 mg sulfur pouch cells Co/Co_0.85_Se@NC catalyst at 100 mA g^–1^. (f) Optical image of a pouch cell powering an energy-saving lamp (permission was obtained for the logo of University of Macau). (g) Structural diagram of the cell. SEM images of the surface of lithium anode after cycling in Li–S cells using (h) Co/Co_0.85_Se@NC-modified separator and (i) blank separator.

In order to evaluate the feasibility of the practical application of the Co/Co_0.85_Se@NC-modified separator in Li–S batteries, a cell with high sulfur loading was fabricated and the electrochemical performance investigated. As shown in Figure 7a, thick C/S cathodes with sulfur mass loadings of 3.6 and 6.2 mg cm^–2^ can deliver initial capacities of 1115 and 916 mAh g^–1^ at 0.1 C, respectively. After 100 cycles, the cells maintain stable capacities of 876 and 713 mAh g^–1^, accounting for 78.6% and 77.8% of the initial capacity, respectively. The rate performance depicted in Figure 7b revealed that the cathodes with a sulfur loading of 5.2 mg cm^–2^ can deliver discharging capacities of 946, 663, and 558 mAh g^–1^ at 0.1, 0.2, and 0.5 C, respectively. Moreover, as shown in Figure S33, the discharge curves at different rates show two distinct voltage platforms, indicating fast reaction kinetics over the Co/Co_0.85_Se@NC DTB sites. Even with higher sulfur loadings of 8.2 and 10.7 mg cm^–2^ under lean electrolyte conditions, corresponding to E/S ratios of 6.6 and 5.8 μL mg^–1^, respectively, the cathodes demonstrate excellent performance, delivering high areal capacities of 8.1 and 10 mAh cm^–2^ (Figure 7c). Notably, these values are significantly superior to the areal capacity (approximately 4 mAh cm^–2^) of commercial Li-ion batteries. We summarize several recently reported performance results from other Li–S cells, which focused on the optimization of the cell configuration through functional sites (Table S2). Our Li–S cells, based on a separator configuration with DTB site design, represent a significant advancement in terms of the decay rate, specific energy under high rate, and cycle stability (Figure 7d and Figure S34). The as-prepared 400 mg level pouch cell was cycled at a current density of 100 mA g^–1^, which still achieves a stable cycle life (Figure 7e). An energy-saving lamp can also be lit up by the pouch cells as shown in Figure 7f, further confirming the potential for practical applications of the as-prepared DTB site materials for advanced Li–S batteries. Furthermore, to demonstrate the effectiveness of Co/Co_0.85_Se@NC in preventing lithium metal corrosion, we conducted an SEM analysis of the solid electrolyte interphase (SEI) on the anode surface after cycling. As shown in Figure 7g–i and Figure S35, the SEI layer on the surface of the lithium anode in the blank separator cell displays visible roughness due to the shuttle effect, whereas the Li anode in the Co/Co_0.85_Se@NC-modified separator cell shows a smooth SEI layer. This observation provides further evidence of the superior performance of the optimized separator in mitigating the polysulfide shuttle effect.

## Conclusion

In summary, the MOF-derived regular dodecahedral mesoporous conductive framework embedding DTB sites of polar Co_0.85_Se and Co@NC clusters has been demonstrated as a promising interlayer for Li–S batteries. Such a multifunctional separator combines the merits of strong binding affinity through Co–S bonds by Co metal catalysis and Li–Se bonds by polar Co_0.85_Se sites with LiPSs, a short ion-transport path, and abundant catalytically active sites to accomplish a highly efficient LiPS confinement-catalysis process, thereby achieving almost no shuttle effect and fast redox reaction kinetics of LiPSs. Hence, excellent performance of Li–S batteries is obtained. As expected, the Li–S cells with a Co/Co_0.85_Se@NC-modified separator exhibit a high reversibility of 932 mAh g^–1^ after 200 cycles at 0.5 C (78% capacity retention), high rate capabilities of 940 and 849 mAh g^–1^ at 2 and 3 C, respectively, and an ultralow decay rate of 0.042% per cycle over 1000 cycles at 2 C. This work demonstrates a strategy for preparing a functional interlayer, constructing a bidirectional catalytic heterojunction and DTB sites based on MOFs, which not only offer guides in the future development of multifunctional interlayer for advanced LSBs but are also expected to be commercialized in Li–S batteries.

## Experimental Section

### Fabrication of Bimetallic Zn/Co-MOF Precursor

The synthesis process is shown in Figure 2d. The chemicals were used as they were received, without any further purification steps. An aqueous methanol solution of 2-methylimidazole (90 mL, 3.94 g) (Sigma-Aldrich) was quickly added to another aqueous methanol solution (90 mL) containing Co(NO_3_)_2_·6H_2_O (4 mmol) and Zn(NO_3_)_2_·6H_2_O (8 mmol) with a molar ratio of 1:2 under magnetic stirring at room temperature for 5 min. After a 6 h reaction at room temperature, the collected precipitates were subjected to three washes with methanol, centrifuged, and finally dried under a vacuum at 60 °C overnight to obtain the Zn/Co-MOF sample.

### Fabrication of the Co@NC, Co_0.85_Se@NC, and Co/Co_0.85_Se@NC Materials

The as-prepared Zn/Co-MOF was annealed in a tube furnace at 900 °C with a ramp rate of 2 °C min^–1^ under an Ar atmosphere to obtain Co@NC powder. Co_0.85_Se@NC and Co/Co_0.85_Se@NC were synthesized by heating the Co@NC and Se powder separately in boats, with the Se powder placed upstream and the Co@NC placed downstream. This calcination process was carried out at 350 °C for 1 h and then at 600 °C for 2 h under an Ar atmosphere, with a heating rate of 2 °C min^–1^. The ratios of obtained Co@NC powder and Se powder were 1:10 and 1:2, respectively, for the synthesis of Co_0.85_Se@NC and Co/Co_0.85_Se@NC samples.

### Fabrication of Sulfur Cathode Material

To synthesize the sulfur cathode materials, a mixture of commercial Super-P and sublimed sulfur powder with a mass ratio of 3:7 was prepared and transferred into an autoclave filled with an Ar atmosphere. The autoclave was then heated to 155 °C for 12 h, leading to the formation of the desired materials.

### Fabrication of Modified Separators

Using a blade coating method, the modified separators were obtained with the Co/Co_0.85_Se@NC-modified separator as an example. A slurry containing Co/Co_0.85_Se@NC powder, Super-P, and poly(vinylidene fluoride) in a mass ratio of 7:2:1 was coated on a Celgard PP separator and dried overnight in a vacuum oven at 60 °C for 12 h. Co@NC, Co_0.85_Se@NC, and Co/Co_0.85_Se@NC had an area loading of approximately 0.28 mg cm^–2^.

### Characterization of Materials

The morphology of the synthesized samples was analyzed using a field emission scanning electron microscope (FESEM, JEOL JSM-7500FA) to obtain scanning electron microscopy (SEM) images. Transmission electron microscopy (TEM), HRTEM images, and the corresponding EDS elemental mappings were captured using a JEOL JEM-2100 electron microscope operating at 200 kV. Powder X-ray diffraction (PXRD) patterns were obtained using a Rigaku Smartlab 9000W diffractometer with Cu Kα radiation (λ = 0.15418 nm) operating at 40 kV and 200 mA. Raman analysis was conducted on a Micro Raman System (Horiba LABHRev-UV) with 633 nm incident radiation. The specific surface area and pore size distribution were determined by BET and Barrett–Joyner–Halenda (BJH) methods, based on N_2_ adsorption desorption isotherms. These isotherms were obtained at liquid nitrogen temperature (−196 °C) using a Micromeritics ASAP 2020 instrument. X-ray photoelectron spectroscopy (XPS) patterns were collected using a Thermo ESCALAB 250 spectrometer with monochromatic Al Kα radiation as the excitation source. The C/S sample underwent TGA (NETZSCH TG 209 F3) under an Ar atmosphere with a heating rate of 10 °C min^–1^, starting from room temperature to 700 °C.

### Adsorption and Catalysis of Functional Sites

#### Preparation of Li_2_S_6_ Solution

A mixture of Li_2_S (Sigma-Aldrich) and sublimed sulfur (Sigma-Aldrich) in a 1:5 mass ratio was added to a traditional electrolyte composed of 1.0 M LiTFSI in a 1:1 v/v mixture of 1,3-dioxolane and dimethyl ether with 2.0 wt % LiNO_3_. The resulting mixture was subjected to vigorous magnetic stirring at 70 °C for an overnight period, ultimately producing a brownish-red Li_2_S_6_ electrolyte with a concentration of 1 M. To measure LiPS adsorption ability, 20 mg of functional materials was immersed in 5 mL of 2 mM Li_2_S_6_ solutions at room temperature.

#### Assembly and Measurement of Symmetrical Cells

The preparation of a symmetrical battery pole piece involved cutting carbon paper into a 12 mm disk, onto which an ethanol dispersion of Co@NC, Co_0.85_Se@NC, and Co/Co_0.85_Se@NC particles was deposited with a loading of 0.5 mg cm^–2^. After drying, two identical electrodes were integrated into a standard 2023-coin cell, and an electrolyte comprising 40.0 μL of Li_2_S_6_ was added. The performance of the symmetrical battery was evaluated for cyclic voltammetry on Bio-Logic EC-LAB (VMP-300) equipment at a scan rate of 100 mV s^–1^, while the scan range spanned from −1 to 1 V.

##### Li_2_S Nucleation and Decomposition Measurement

To prepare a Li_2_S_8_ electrolyte with a concentration of 0.20 mol L^–1^, sublimed sulfur and Li_2_S were mixed in a molar ratio of 7:1 in tetraglyme. The mixture was then stirred vigorously under an Ar atmosphere at 60 °C for 24 h. To assemble the coin cell, a commercial CP was utilized as a current collector with functional materials of 0.5 mg cm^–2^ dispersed as the cathode and lithium foils as counter electrodes. 25 μL of Li_2_S_8_ electrolyte was used in the CP compartment, while the traditional electrolyte without Li_2_S_8_ (20 μL) was dropped in the anode. To induce Li_2_S nucleation, the cells were discharged galvanostatically at 0.112 mA to 2.06 V and then potentiostatically discharged at 2.05 V until the current decreased below 10^–5^ A. On the other hand, to decompose Li_2_S, the cells were galvanostatically discharged to 1.70 V at 0.112 mA and then potentiostatically charged at 2.35 V until the charge current was less than 10^–5^ A.

### Electrochemical Measurements of Li–S Cells

To conduct the electrochemical tests, coin-type half-cells (2032) were assembled in an argon-filled glovebox. The cathode C/S material was mixed with Super-P and PVDF in a ratio of 8:1:1 using a moderate amount of NMP, stirred to obtain a uniform slurry, which was then coated on an aluminum foil and dried at 60 °C for 12 h. The resulting disk had a diameter of 12 mm. For the half cells and cyclic voltammetry test, the total mass loading was about 1.0 mg cm^–2^. The cells were assembled with lithium metal as the anode and Celgard 2500 as the separator, and the electrolyte used was lithium bis(trifluoromethanesulfonyl)imide (LiTFSI, 1.0 M) in 1,3-dioxolane and 1,2-dimethoxyethane (v/v = 1:1) with 0.2 M LiNO_3_ as an additive. The electrolyte/sulfur ratio for the standard coin cell configuration was approximately 18 μL mg^–1^. For high sulfur loading configurations of 3.6, 5.2, 6.2 , 8.2, and 10.7 mg cm^–2^, the electrolyte/sulfur ratios were 12, 10, 8, 6, and 5.8 μL mg^–1^, respectively. The cells were assembled in an Ar-filled glovebox (Super 1220/750, Mikrouna) with an O_2_ and H_2_O content below 0.01 ppm. The cycle performance and rate performance were evaluated using a Neware Battery Tester with a voltage window of 1.7–2.8 V, and a CV test was conducted with Bio-Logic EC-LAB (VMP-300) equipment at room temperature.

### *In Situ* Measurements of Li–S Cells

#### *In Situ* Raman Spectroscopy

The cells were assembled into an *in situ* Raman device with a quartz window from Beijing Scistar Technology Co. Ltd. To detect the dissolved lithium polysulfides in the electrolyte, small holes of 2 mm were manufactured in the lithium film, allowing the light source to pass through. The Raman raster was set to 2400|mm^–1^, and the wavelength used was 633 nm. During measurement, the cell was discharged at a current of 0.2 C, and the recorded Raman shift spanned from 100 to 500 cm^–1^.

#### *In Situ* XRD Spectroscopy

A viscous slurry was formed by mixing C/S, Super P, and PVDF binder in an NMP solution with a weight ratio of 8:1:1. The slurry was coated onto a thin aluminum film and dried for 12 h. The resulting sulfur loading was approximately 1.8 mg cm^–2^. The cells were then assembled in an *in situ* XRD device. The current rate during measurement was 0.1 C, and the test angle was set in a range of 20–30°.

### Theoretical Calculations

The Vienna ab initio Simulation Package (VASP) was utilized to perform all calculations in spin-polarized density functional theory (DFT). To handle ion–electron interactions, a projector augmented wave pseudopotential was employed, while a plane-wave cutoff energy of 450 eV was used to ensure accuracy. The Perdew–Burke–Ernzerhof (PBE) exchange-correlation function was chosen to calculate the exchange-correlation interactions. The graphene supercell was set to 6 × 6 × 1 with a vacuum distance of about 15 Å to ensure a negligible interaction. The Co@NC supercell had 64, 4, and 9 Co atoms. The Brillouin zones were sampled by the 2 × 2 × 1 Γ centered *k*-point mesh for calculating surface properties. All the atoms were allowed to relax until the residual force below −0.03 eV Å^–1^. The binding energy was determined using the equation
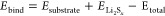
where *E*_bind_ denotes the binding energy, *E*_substrate_ represents the total energy of the optimized surface, *E*_Li_2_S_*x*__ is the total energy of the optimized Li_2_S_*x*_, and *E*_total_ represents the total energy of the optimized surface after adsorbing Li_2_S_*x*_.

## Supporting Information Available

Additional data including characterizations (SEM, XRD, Raman, XPS), electrochemical properties, DFT calculations, and fabricated flexible devices (PDF)
