# Supplementary material for: Interfacial “Double-Terminal Binding Sites” Catalysts Synergistically Boosting the Electrocatalytic Li2S Redox for Durable Lithium–Sulfur Batteries
Source: ACS Nano. 2024 Mar 11;18(12):8839–52. doi: 10.1021/acsnano.3c11903 (PMC10976959; doi:10.1021/acsnano.3c11903)
Supplement: Supplementary file 1 — nn3c11903_si_001.pdf [file nn3c11903_si_001.pdf]

SUPPORTING INFORMATION

Interfacial "Double-Terminal Binding Sites" Catalysts Synergistically Boosting the Electrocatalytic Li_2S Redox for Durable Lithium-Sulfur Batteries

Huifang Xu,‡ Qingbin Jiang,‡ Kwan San Hui, Shuo Wang, Lingwen Liu, Tianyu Chen, Yunshan Zheng, Weng Fai Ip, Duc Anh Dinh, Chenyang Zha, Zhan Lin,* Kwun Nam Hui**

H. Xu, Q. Jiang, S. Wang, L. Liu, T. Chen, Y. Zheng, Prof. C. Zha, Prof. K. N. Hui
Joint Key Laboratory of the Ministry of Education, Institute of Applied Physics and Materials Engineering, University of Macau, Avenida da Universidade, Taipa, Macau SAR, China, E-mail: bizhui@um.edu.mo

Weng Fai Ip
Department of Physics and Chemistry, Faculty of Science and Technology, University of Macau, 999078, PR China

Duc Anh Dinh
NTT Hi-Tech Institute, Nguyen Tat Thanh University, Ho Chi Minh City 700000, Vietnam

Prof. K. S. Hui
School of Engineering, Faculty of Science, University of East Anglia, Norwich, NR4 7TJ, United Kingdom, E-mail: k.hui@uea.ac.uk

Prof. Z. Lin
School of Chemical Engineering and Light Industry, Guangdong University of Technology, Guangzhou, 510006, China, E-mail: zhanlin@gdut.edu.cn

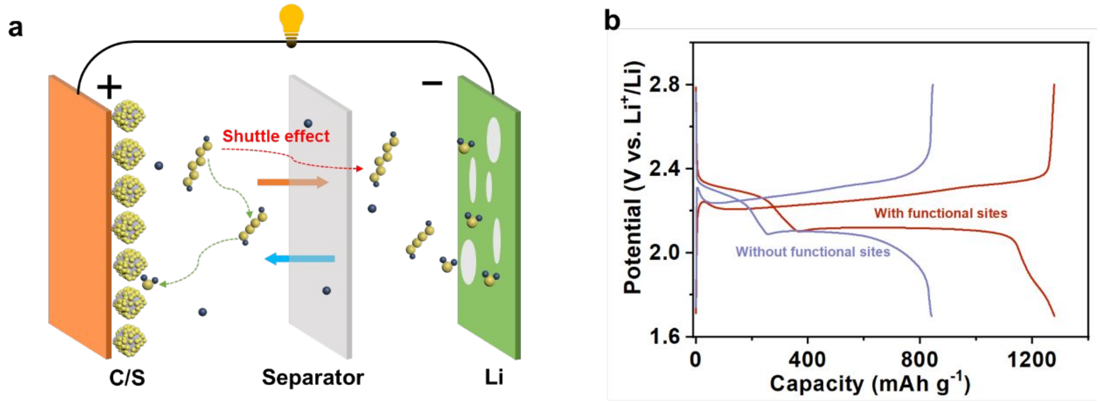

Figure S1. a) Schematic illustrations of the shuttle effect in a typical Li-S battery. b) The importance of functional sites in a typical Li-S battery.

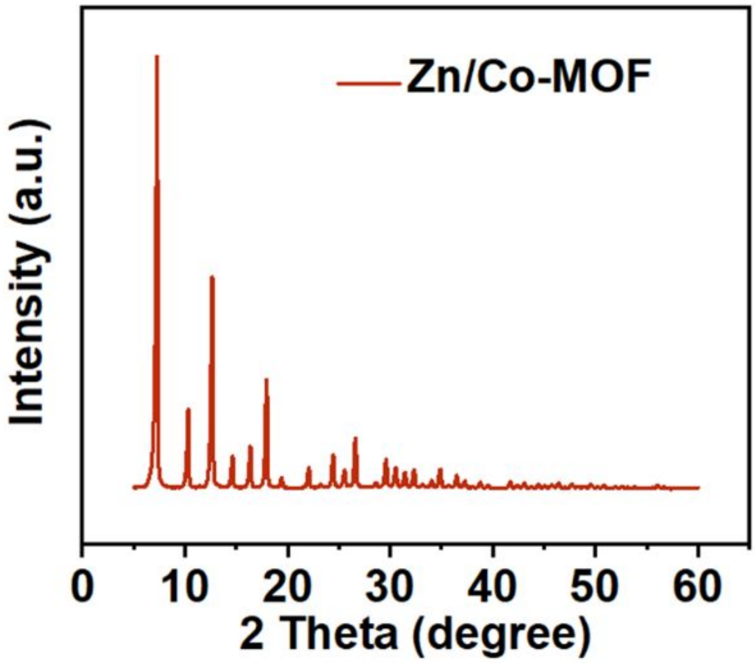

Figure S2. XRD pattern of Zn/Co-MOF.

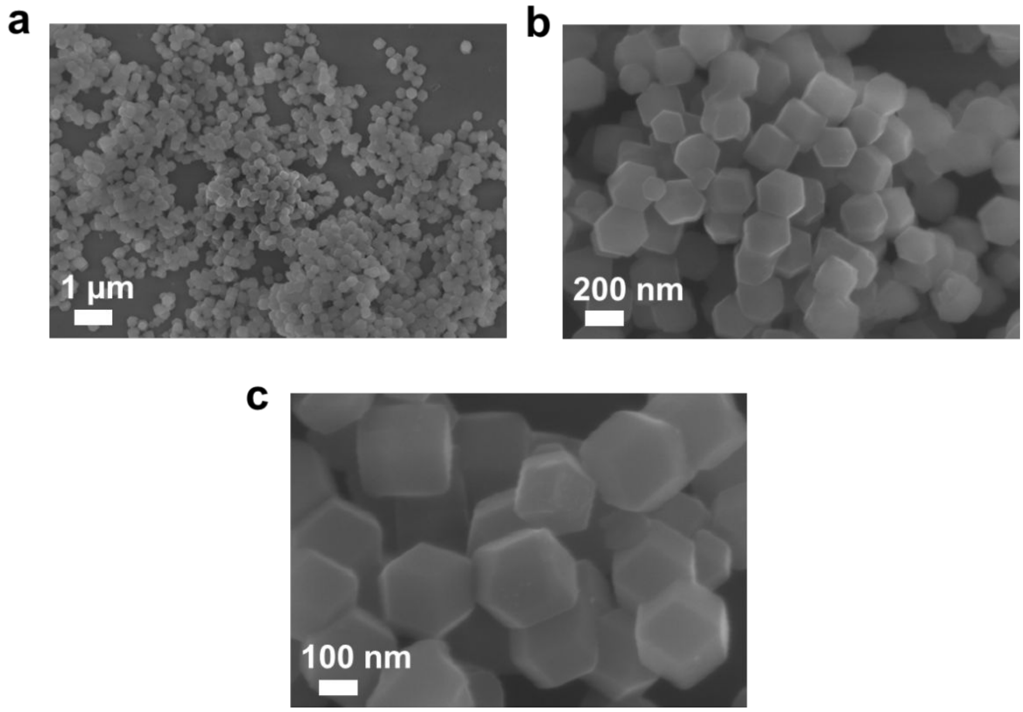

Figure S3. a-c) SEM images of Zn/Co-MOF.

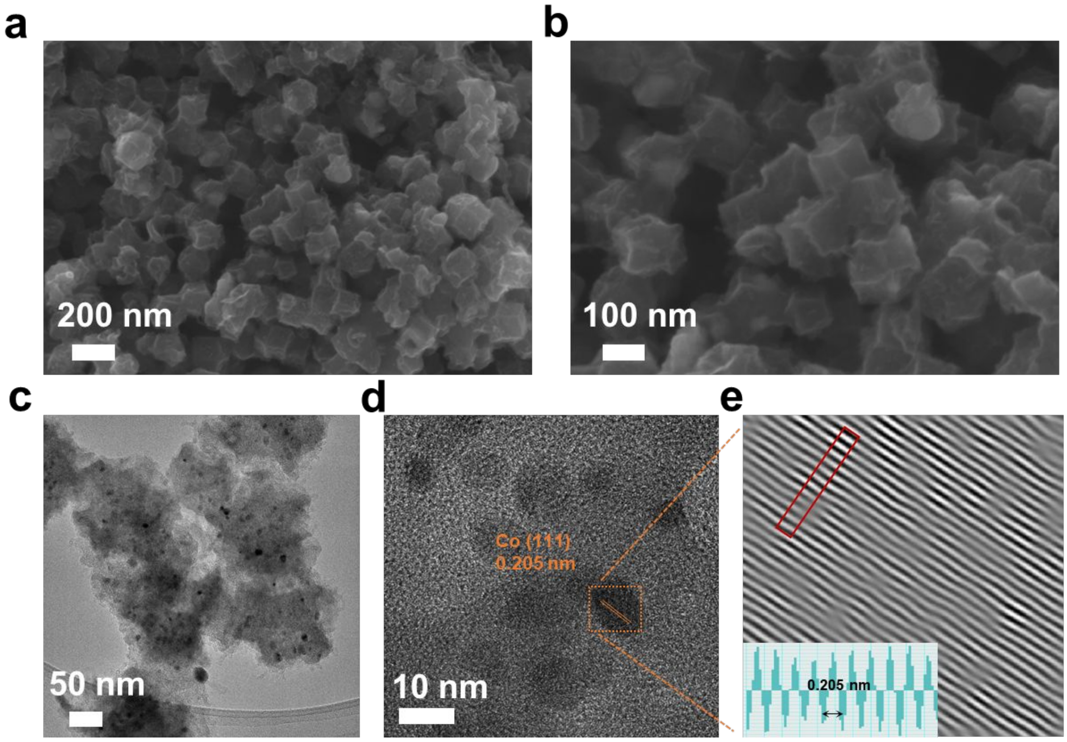

Figure S4. a,b) SEM images of Co@NC, c) TEM image, and d) high-resolution TEM image of Co@NC, e) IFFT lattice image of the selected area of Co@NC (Inset: lattice distance profiles of the area in red).

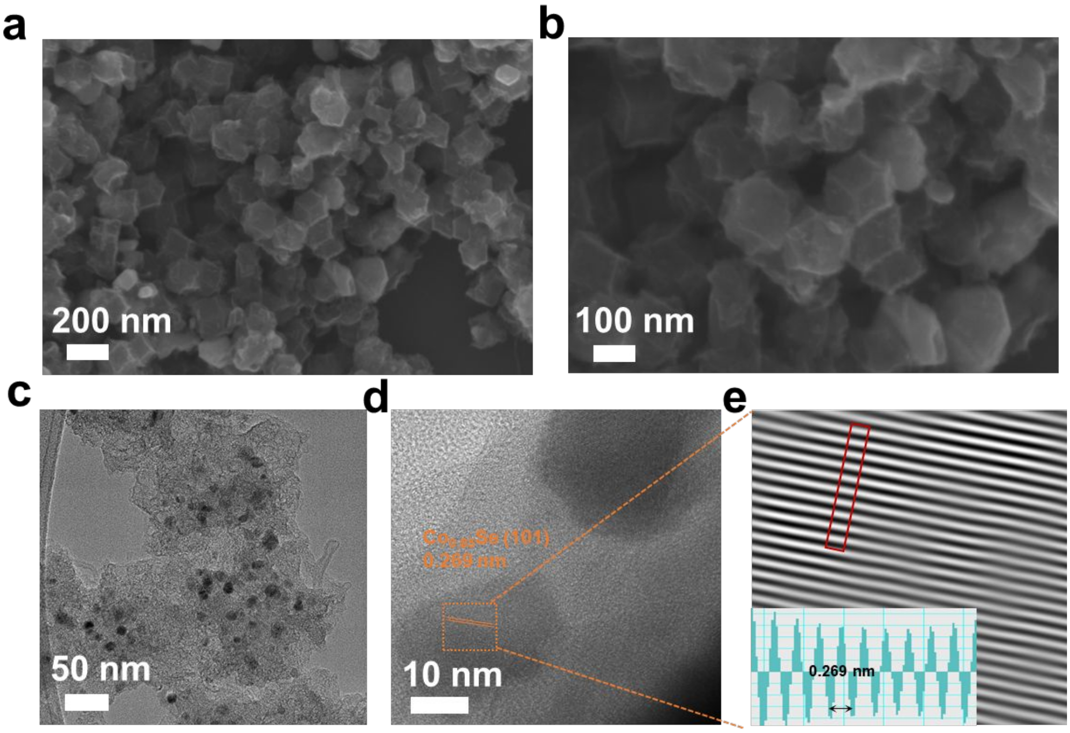

Figure S5. a,b) SEM images of $Co_{0.85}Se$@NC, c) TEM image, and d) high-resolution TEM image of $Co_{0.85}Se$@NC, e) IFFT lattice image of the selected area of $Co_{0.85}Se$@NC (Inset: lattice distance profiles of the area in red).

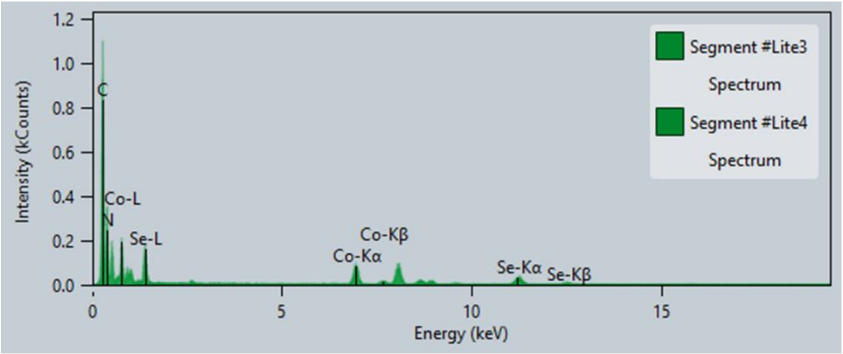

Figure S6. EDS spectrum of Co/$Co_{0.85}$Se@NC.

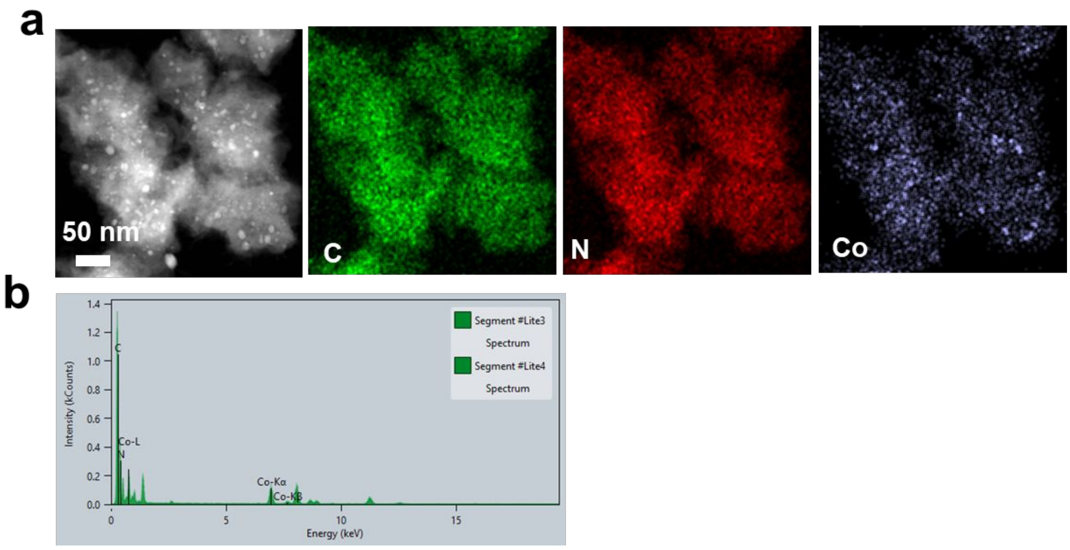

Figure S7. a) TEM-EDX element mapping of the Co@NC, b) EDS spectrum of Co@NC.

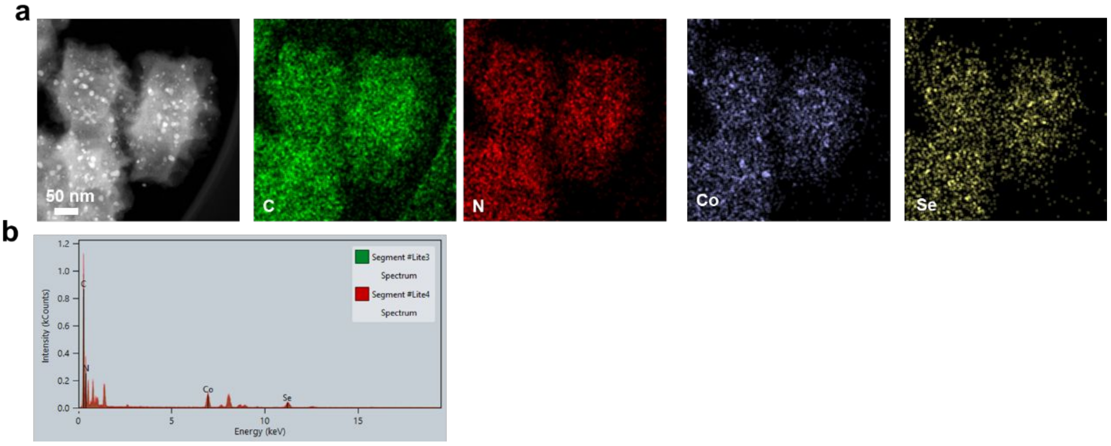

Figure S8. a) TEM-EDX element mapping of the $Co_{0.85}Se$@NC, b) EDS spectrum of $Co_{0.85}Se$@NC.

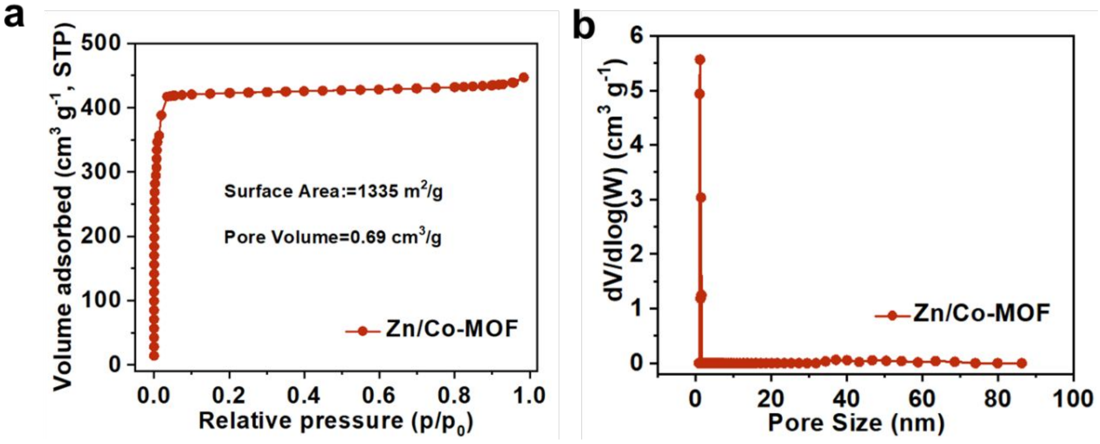

Figure S9. a) Nitrogen adsorption–desorption isotherm, and b) pore size distributions of Zn/Co-MOF.

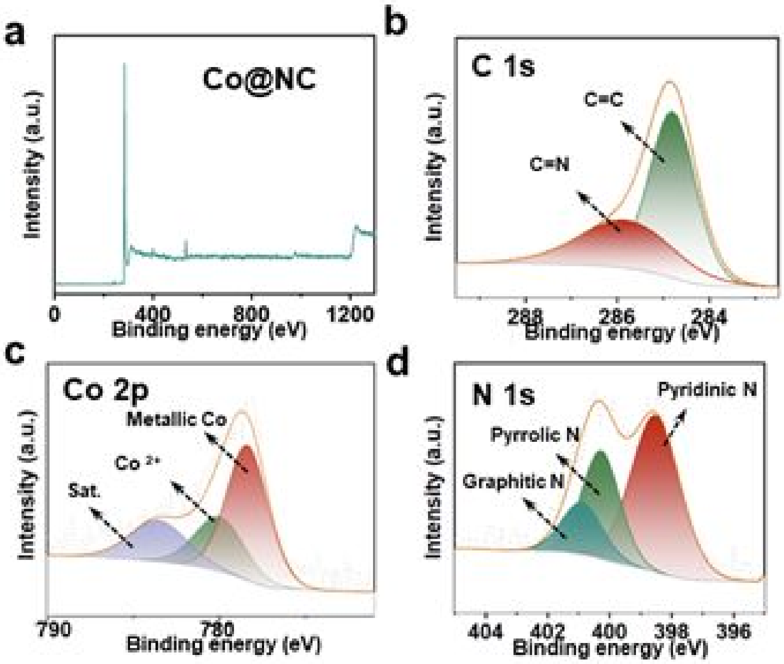

Figure S10. a) X-ray photoelectron spectroscopy (XPS) spectra of Co@NC, high-resolution XPS plots of b) C 1s, c) Co 2p, and d) N 1s of Co@NC.

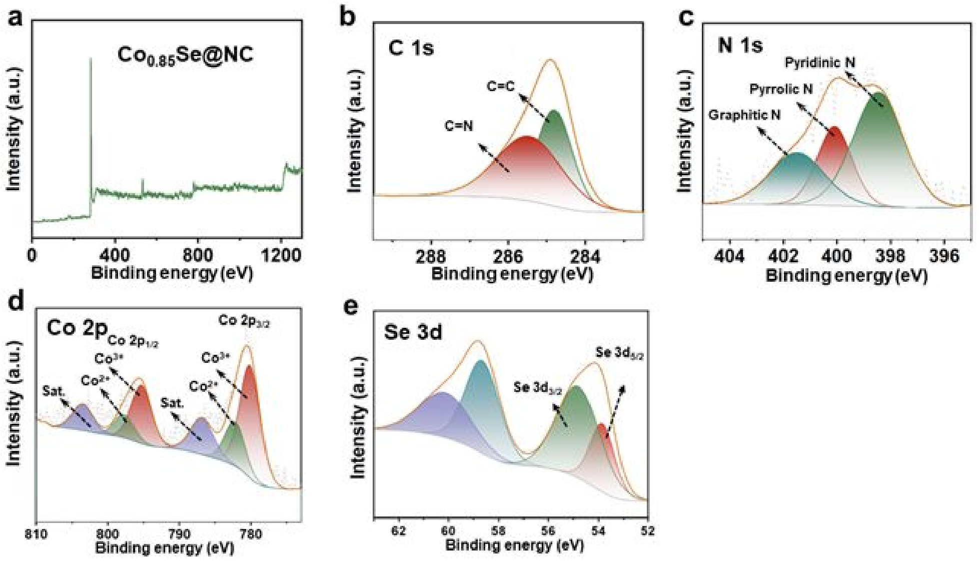

Figure S11. a) X-ray photoelectron spectroscopy (XPS) spectra of $Co_{0.85}Se$@NC, high-resolution XPS plots of b) C 1s, c) N 1s, and d) Co 2p, e) Se 3d of $Co_{0.85}Se$@NC.

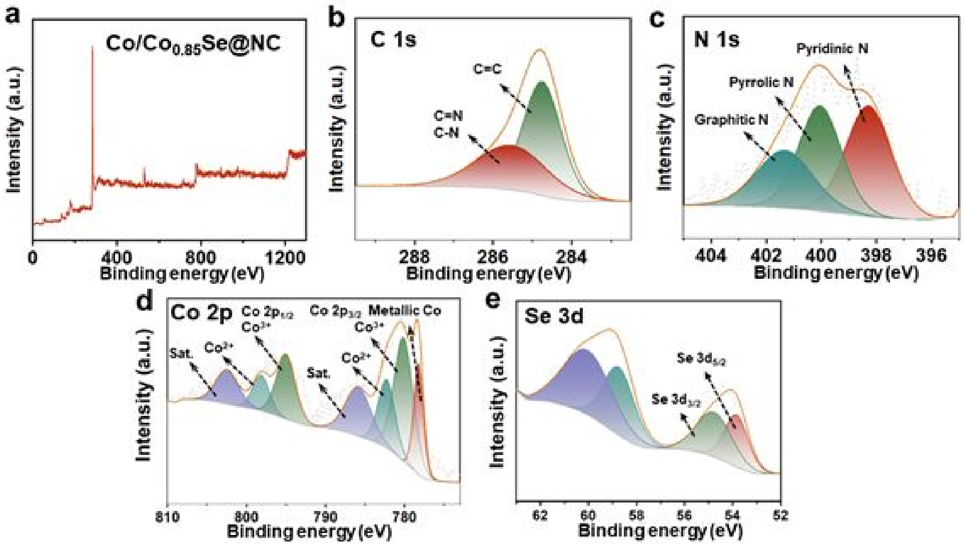

Figure S12. a) X-ray photoelectron spectroscopy (XPS) spectra of $Co/Co_{0.85}Se@NC$, high-resolution XPS plots of b) C 1s, c) N 1s, and d) Co 2p, e) Se 3d of $Co/Co_{0.85}Se@NC$.

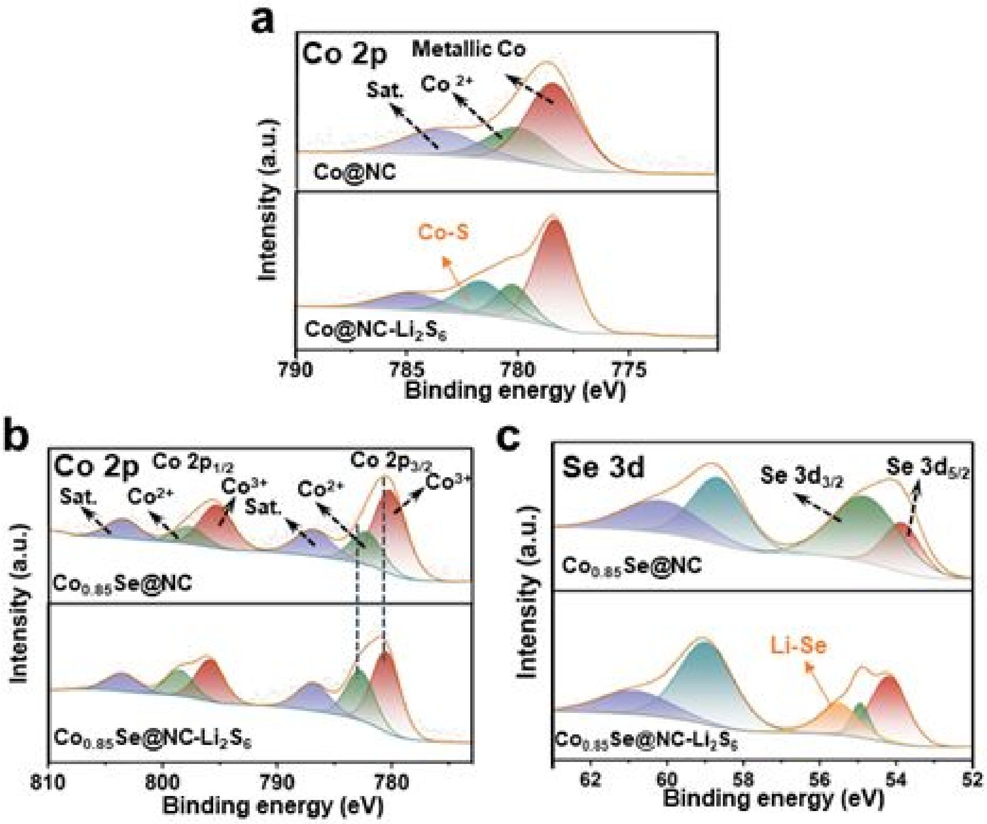

Figure S13. a) High-resolution XPS plots of Co 2p of Co@NC before and after adsorption of Li_2S_6. b) High-resolution XPS plots of Co 2p of $Co_{0.85}Se$@NC before and after adsorption of Li_2S_6. c) High-resolution XPS plots of Se 3d of $Co_{0.85}Se$@NC before and after adsorption of Li_2S_6.

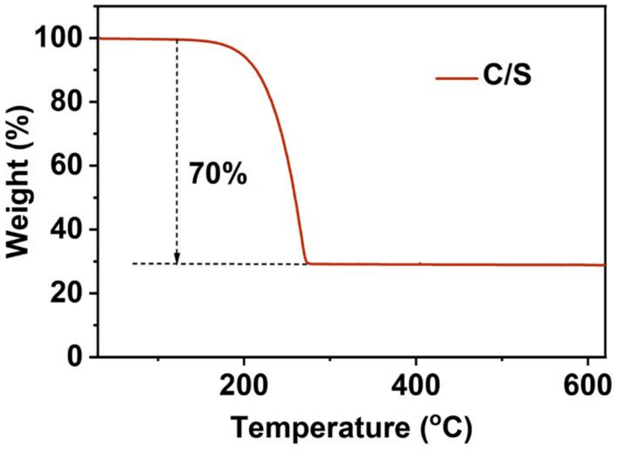

Figure S14. TGA curves of C/S composite at a heating rate of 10 °C min^{-1} under Ar atmosphere.

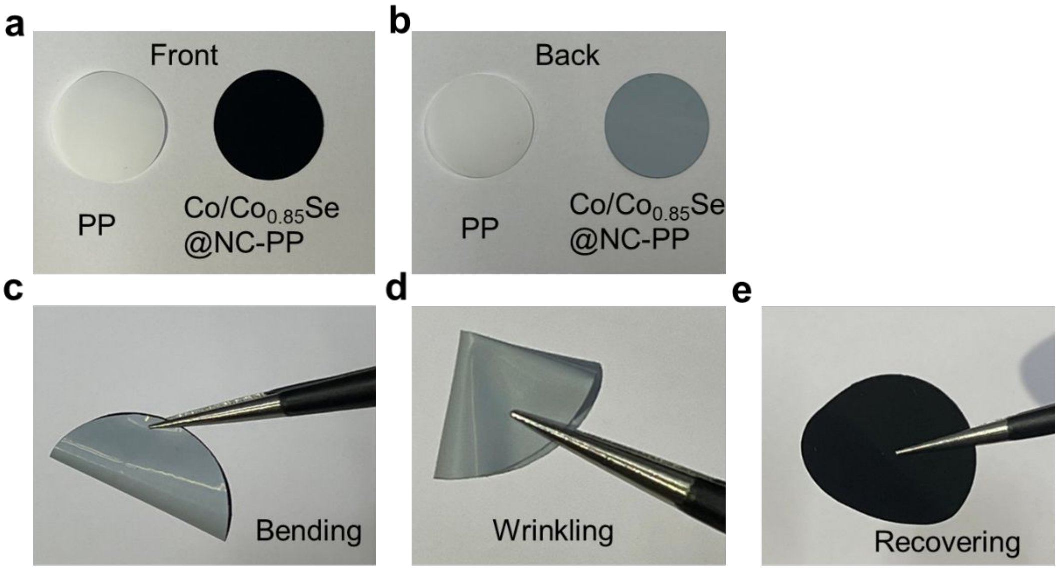

Figure S15. Digital photos of the modified separator. a,b) the front and back, and c-e) under various mechanical stresses.

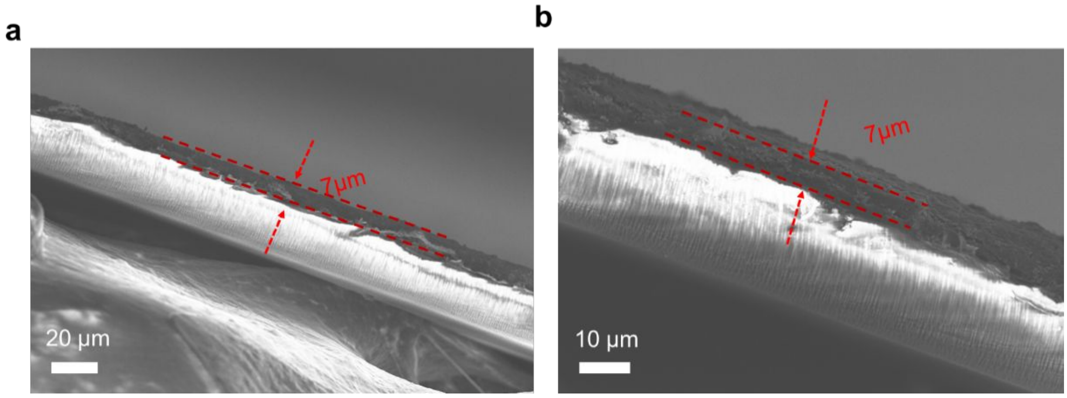

Figure S16. a,b) SEM images of the Side-view of the modified separator.

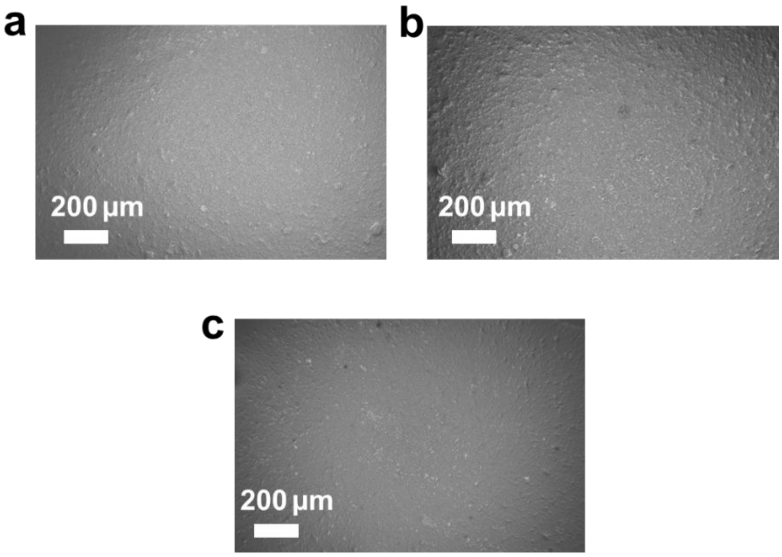

Figure S17. SEM images of Co@NC, $Co_{0.85}Se$@NC, and Co/$Co_{0.85}Se$@NC modified separators.

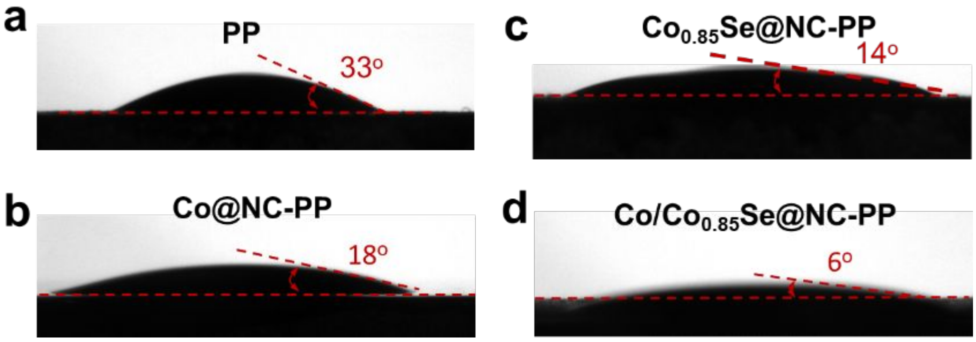

Figure S18. The DME/DOL electrolyte contact angle shots of the PP, Co@NC-PP, $Co_{0.85}Se$@NC-PP, and Co/$Co_{0.85}Se$@NC-PP.

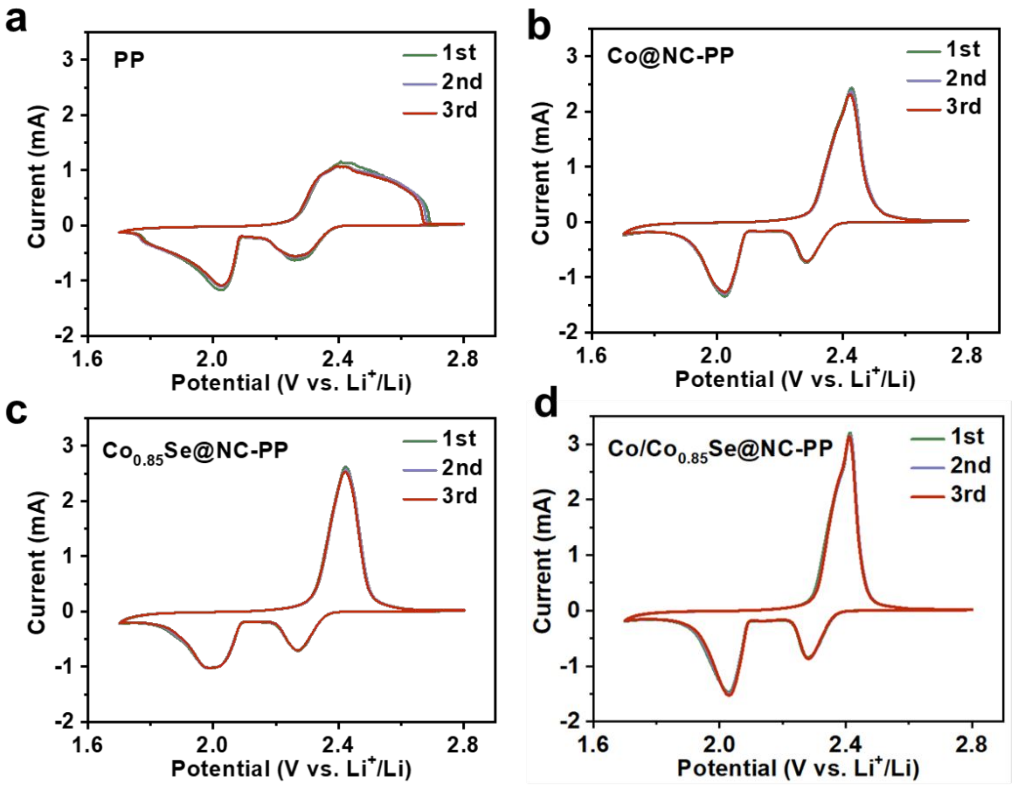

Figure S19. CV profiles of Li-S cells with a) Blank PP, b) Co@NC-modified, c) $Co_{0.85}Se$@NC-modified, and d) Co/$Co_{0.85}Se$@NC-modified separators.

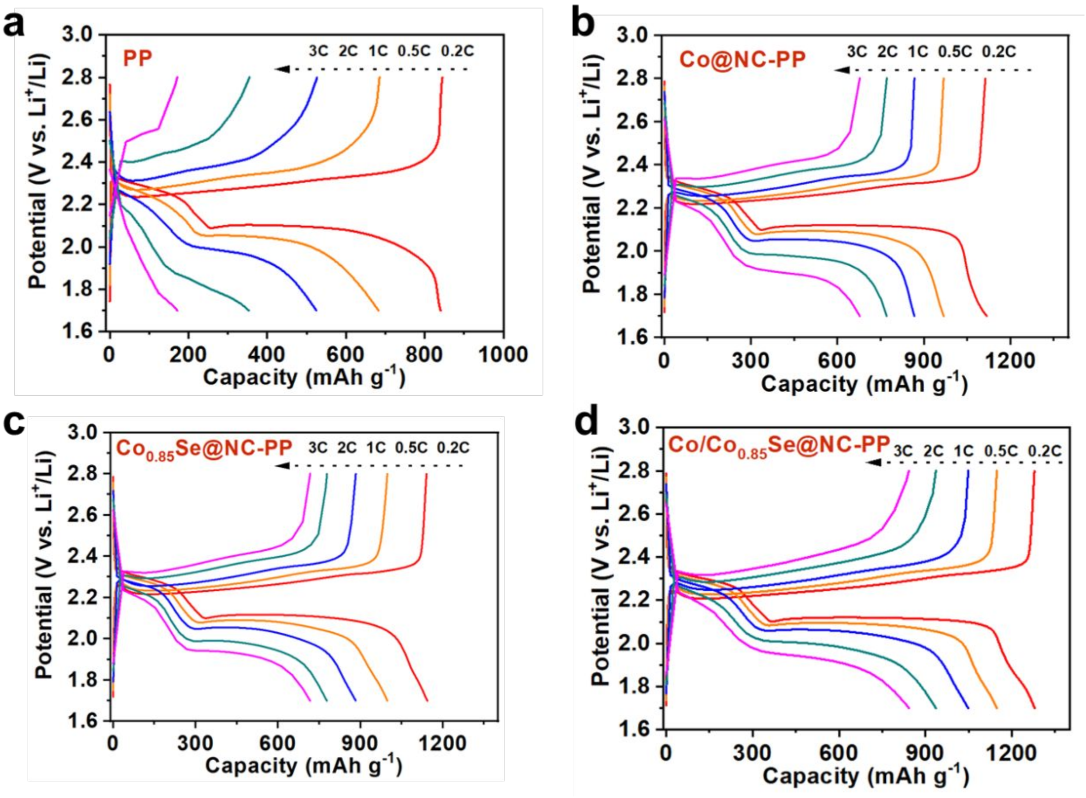

Figure S20. Charge/discharge profiles of Li–S cells with different separators under various current densities. a) PP separator, b) Co@NC-modified separator, c) $Co_{0.85}Se$@NC-modified separator, and d) Co/$Co_{0.85}Se$@NC-modified separator.

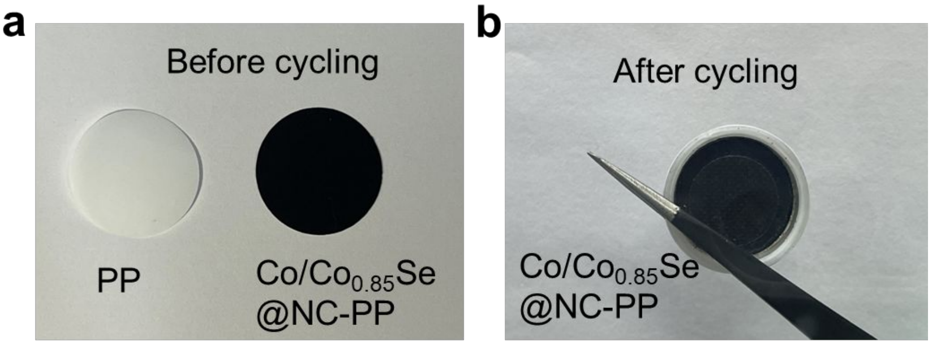

Figure S21. The digital images of Co/$Co_{0.85}$Se@NC-PP before and after cycling.

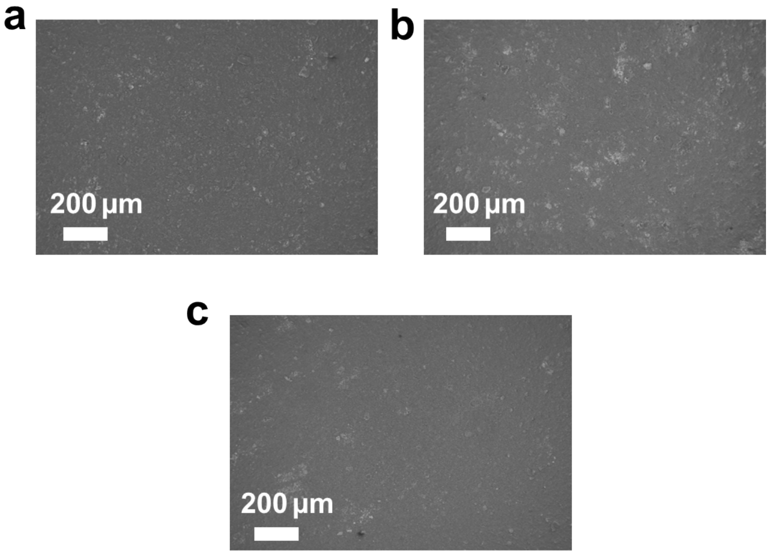

Figure S22. SEM images of Co@NC, $Co_{0.85}$Se@NC, and Co/$Co_{0.85}$Se@NC modified separators after cycling.

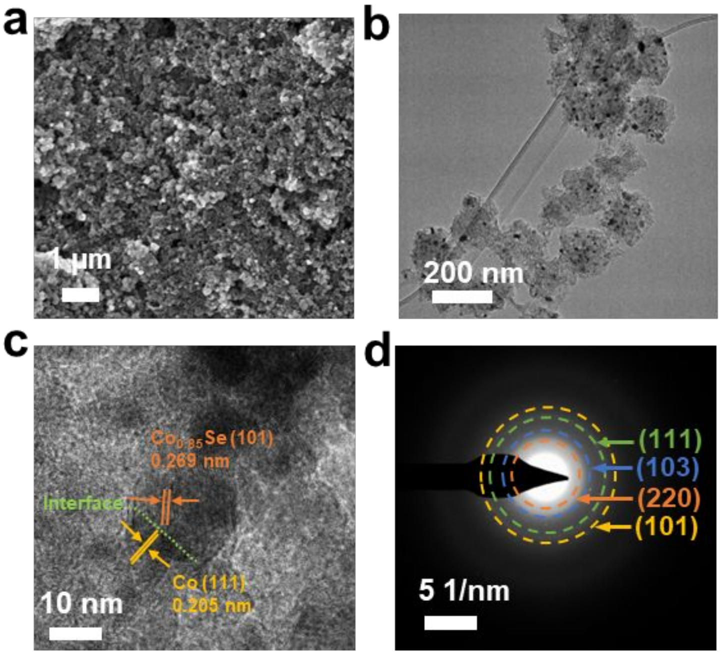

Figure S23. a) SEM image, b) TEM images, c) High-resolution TEM image, and d) SAED pattern of Co/$Co_{0.85}$Se@NC after cycling.

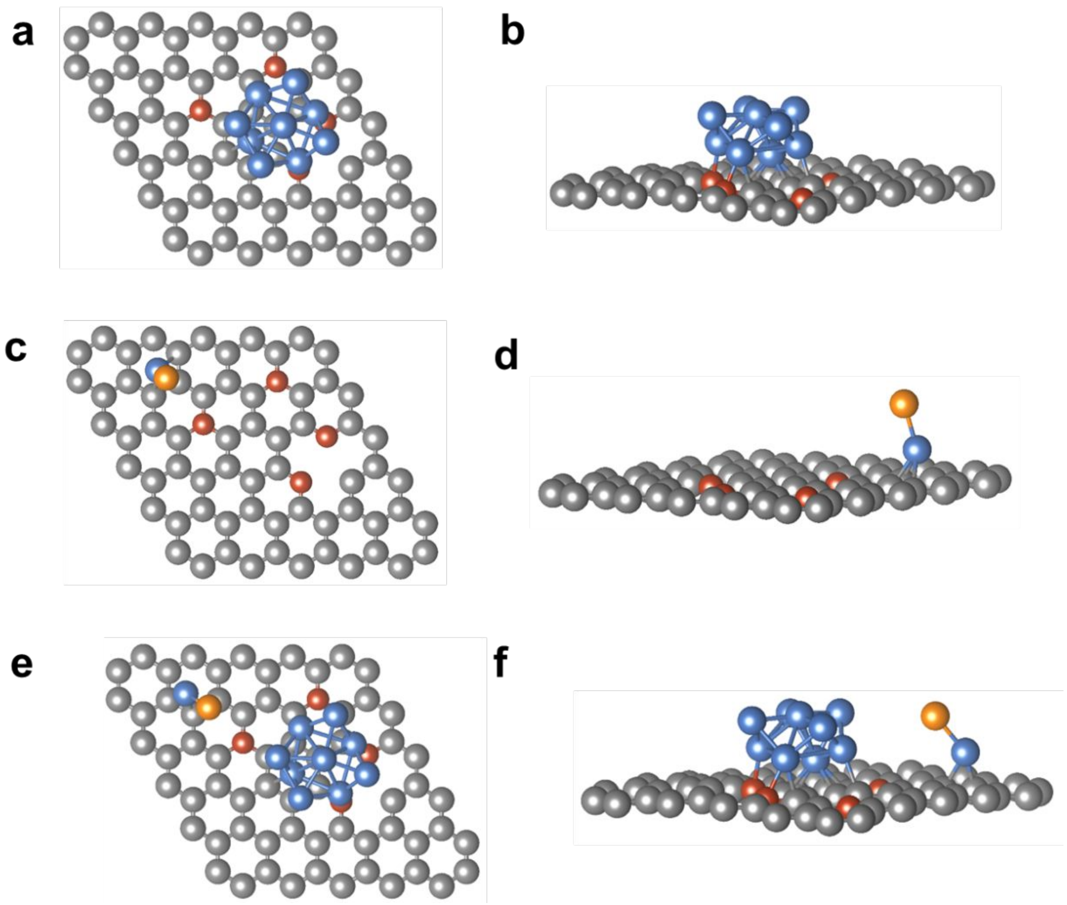

Figure S24. Top view and side view of optimized structures of a,b) Co@NC, c,d) $Co_{0.85}Se$@NC, and e,f) Co/$Co_{0.85}Se$@NC.

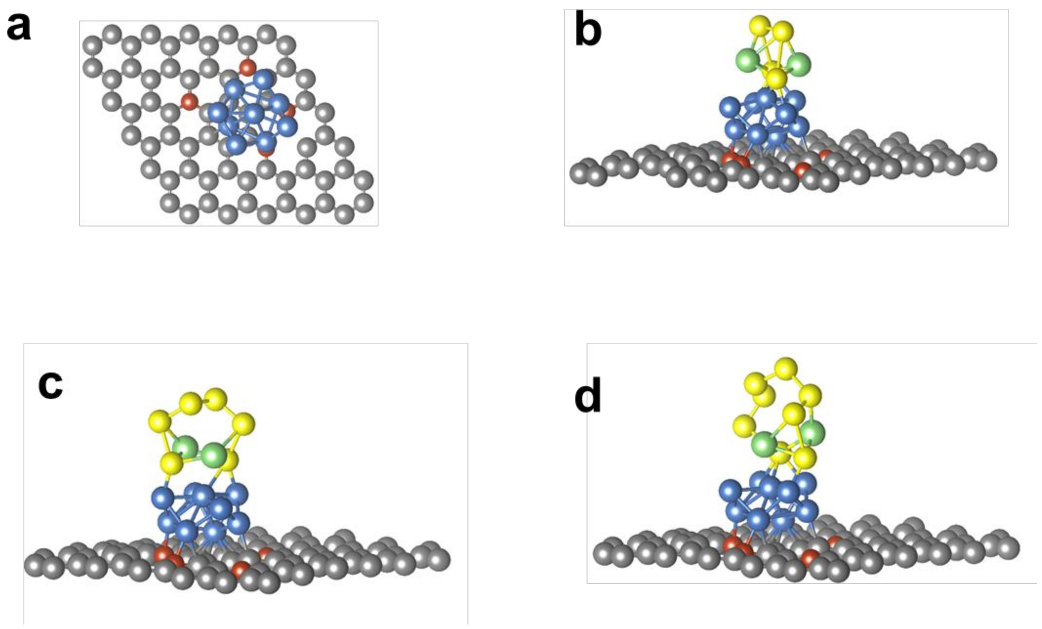

Figure S25. The optimized adsorption structures of b) Li_2S_4, c) Li_2S_6, d) Li_2S_8 on the Co@NC surface.

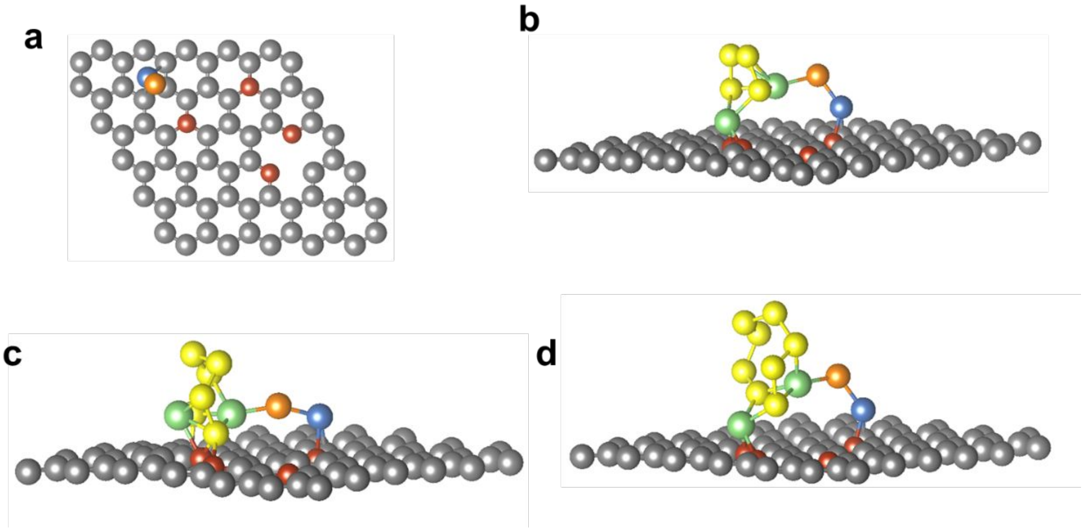

Figure S26. The optimized adsorption structures of b) Li_2S_4, c) Li_2S_6, d) Li_2S_8 on $Co_{0.85}Se$@NC surface.

SRR process		$S_8 + 16Li^+ + 16e^- = 8Li_2S$
$\Delta G1$	S_8	$S_8 + * = S_8*$
$\Delta G2$	Li_2S_8	$S_8 + * + 2Li^+ + 2e^- = Li_2S_8*$
$\Delta G3$	Li_2S_6	$Li_2S_8* + 1/3* + 2/3Li^+ + 2/3e^- = 4/3\ Li_2S_6*$
$\Delta G4$	Li_2S_4	$4/3Li_2S_6* + 2/3* + 4/3Li^+ + 4/3e^- = 2Li_2S_4*$
$\Delta G5$	Li_2S_2	$2Li_2S_4* + 2* + 4Li^+ + 4e^- = 4Li_2S_2*$
$\Delta G6$	Li_2S	$4Li_2S_2* + 4* + 8Li^+ + 8e^- = 8Li_2S*$

Figure S27. Two–electrons reaction equations of Li-S batteries.

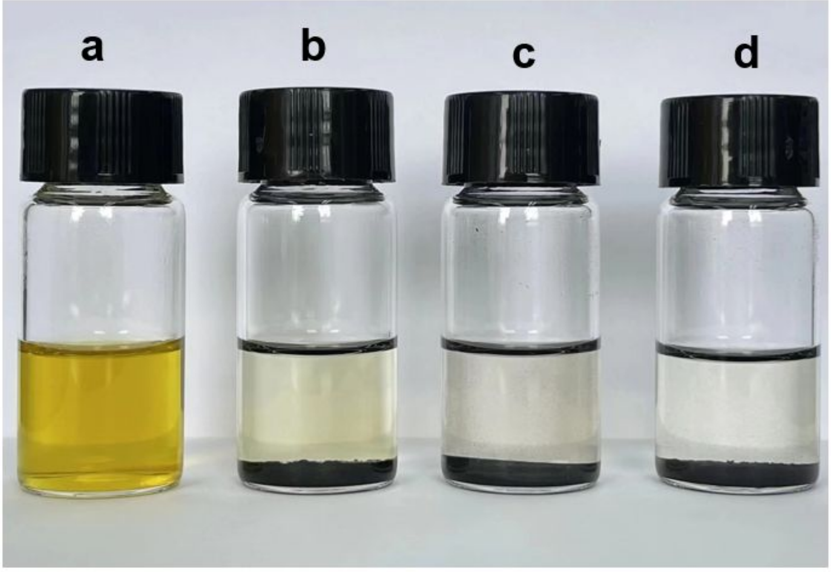

Figure S28. Visualized images of a) pure Li_2S_6 solution and Li_2S_6 solutions after adding b) Co@NC, c) $Co_{0.85}Se$@NC, and d) Co/$Co_{0.85}Se$@NC powder.

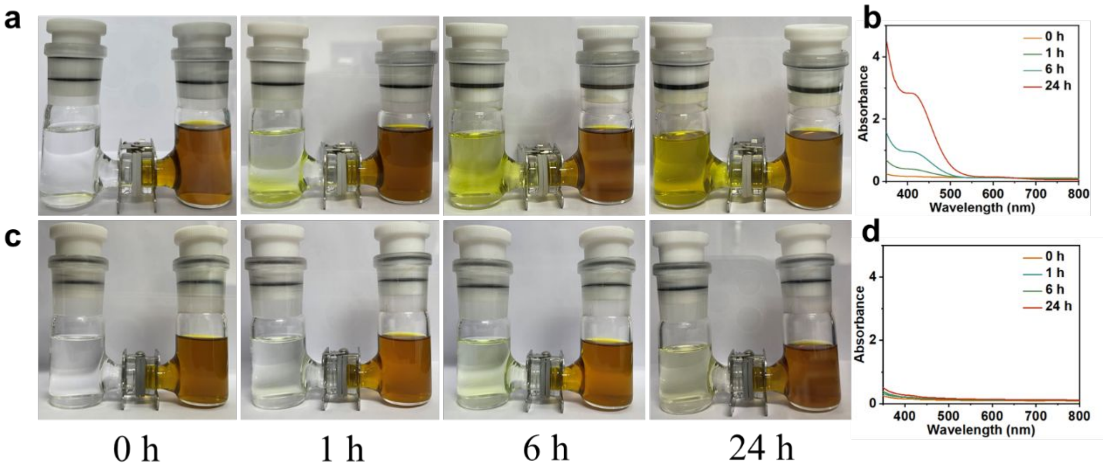

Figure S29. Polysulfides permeation tests and corresponding UV-Vis absorption spectra in the right compartment of the H-typed glass cells with a,b) blank separator, and c,d) $Co/Co_{0.85}Se$@NC-modified separator.

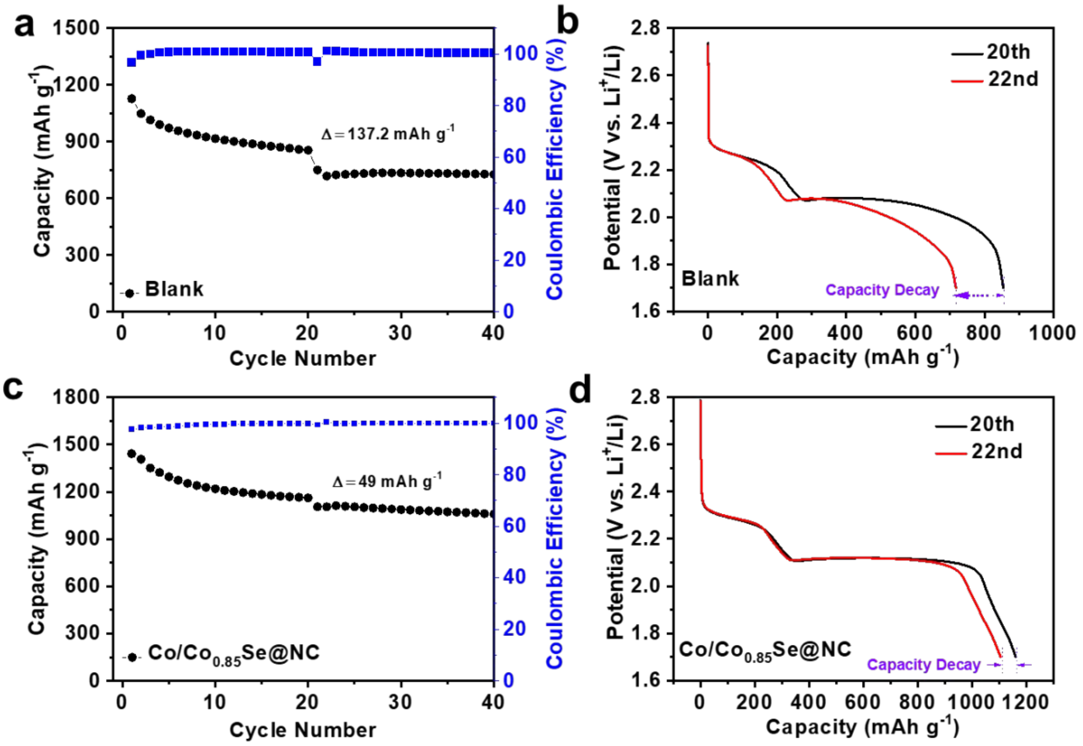

Figure S30. Self-discharge test of Li-S cells with a) blank separator, and c) $Co/Co_{0.85}Se@NC$-modified separator at 0.2 C (after 20th continuous cycle, 7-day rest at 2.1 V during discharge process in 21 cycling). a, c) Corresponding cycling performance of the cells, and b, d) discharge profiles of the 20th and 22nd cycle of the two cells.

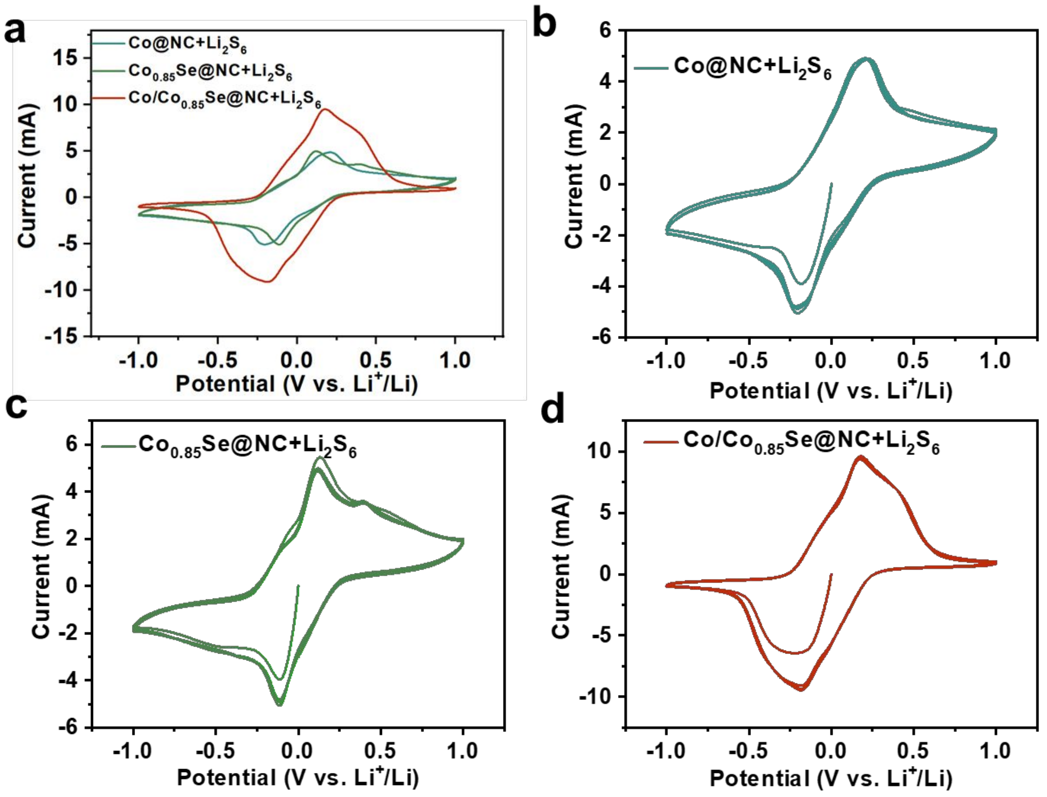

Figure S31. CV profiles of the Li_2S_6 symmetric cells assembled using a) three catalysts, b) Co@NC, c) $Co_{0.85}Se$@NC, and d) Co/$Co_{0.85}Se$@NC as electrodes at a scan rate of 100 mV s^{-1}.

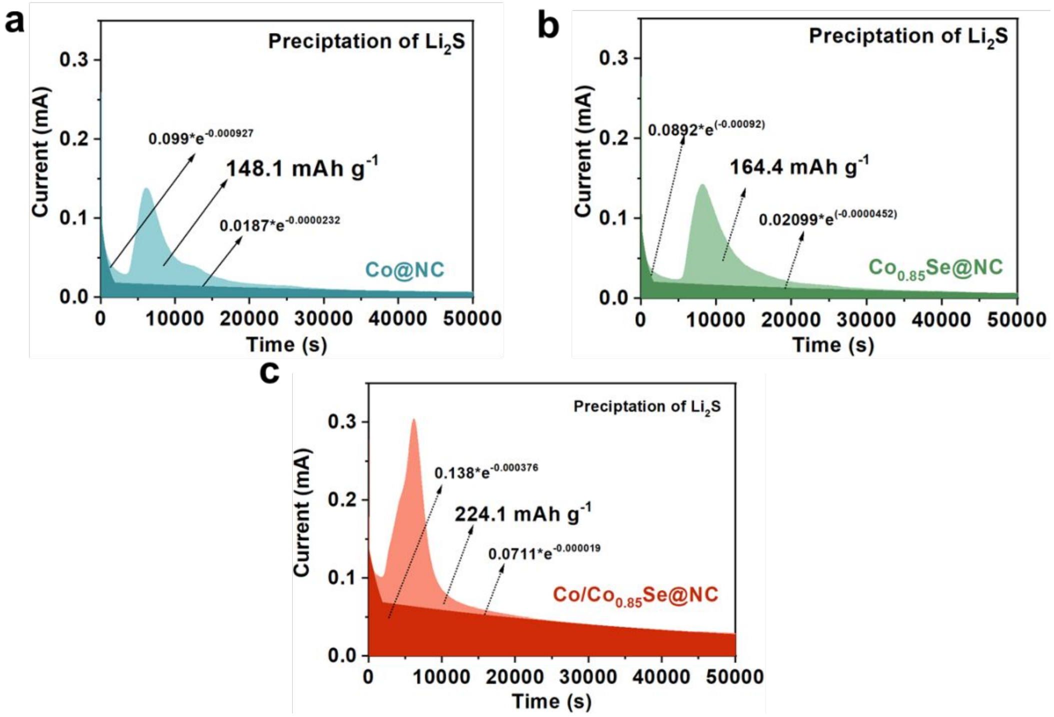

Figure S32. Current vs. time curve for potentiostatic discharge at 2.05 V on a) Co@NC, b) $Co_{0.85}Se$@NC, and c) Co/$Co_{0.85}Se$@NC surfaces**.**

An experiment was conducted to investigate the growth rates of Li_2S on different surfaces during potentiostatic discharge at 2.05 V. The cells were initially galvanostatically discharged to 2.06 V and then kept at a potentiostatic state of 2.05 V to allow for Li_2S nucleation. Faraday's Law was used to fit the data. The resulting current vs. time curve was fitted using the integration of two exponentially decaying curves, representing the reduction of Li_2S_6 and Li_2S_8, respectively.

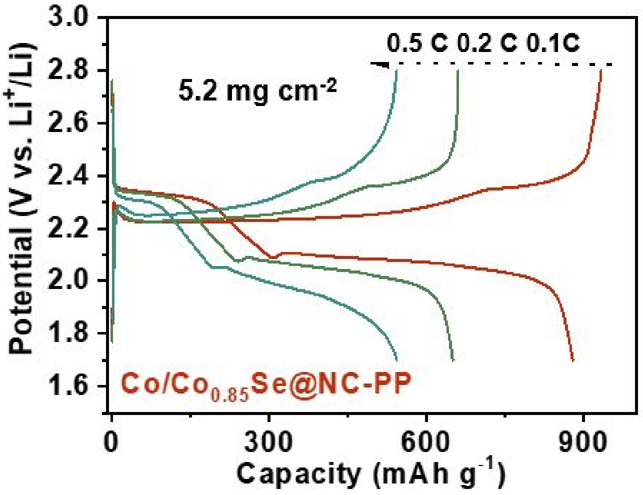

Figure S33. Charge/discharge profiles of Li-S cell with $Co/Co_{0.85}Se$@NC-modified separator under high sulfur loadings at various current densities.

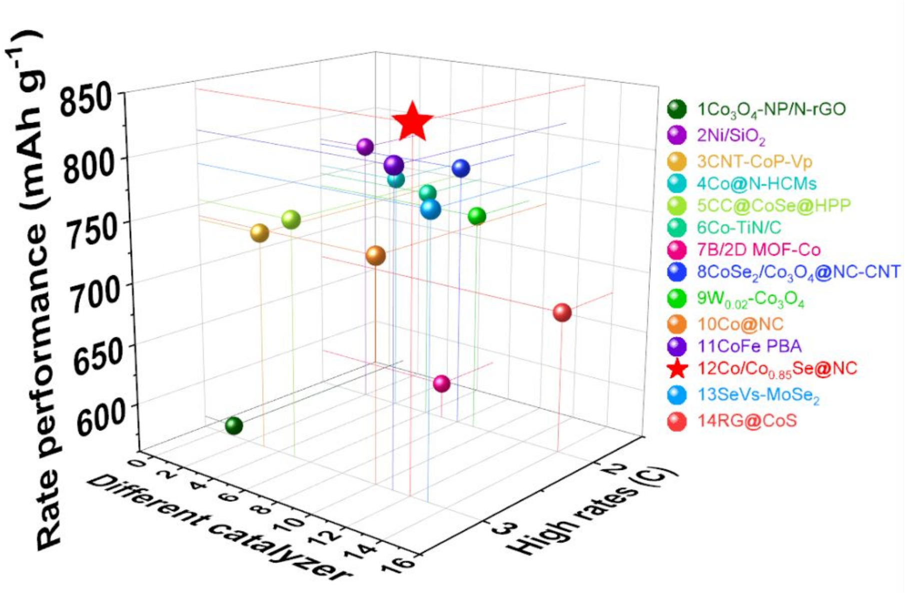

Figure S34. Cell performance comparison between our Li-S cells with DTB sites modified separator and previously reported Li-S cells.

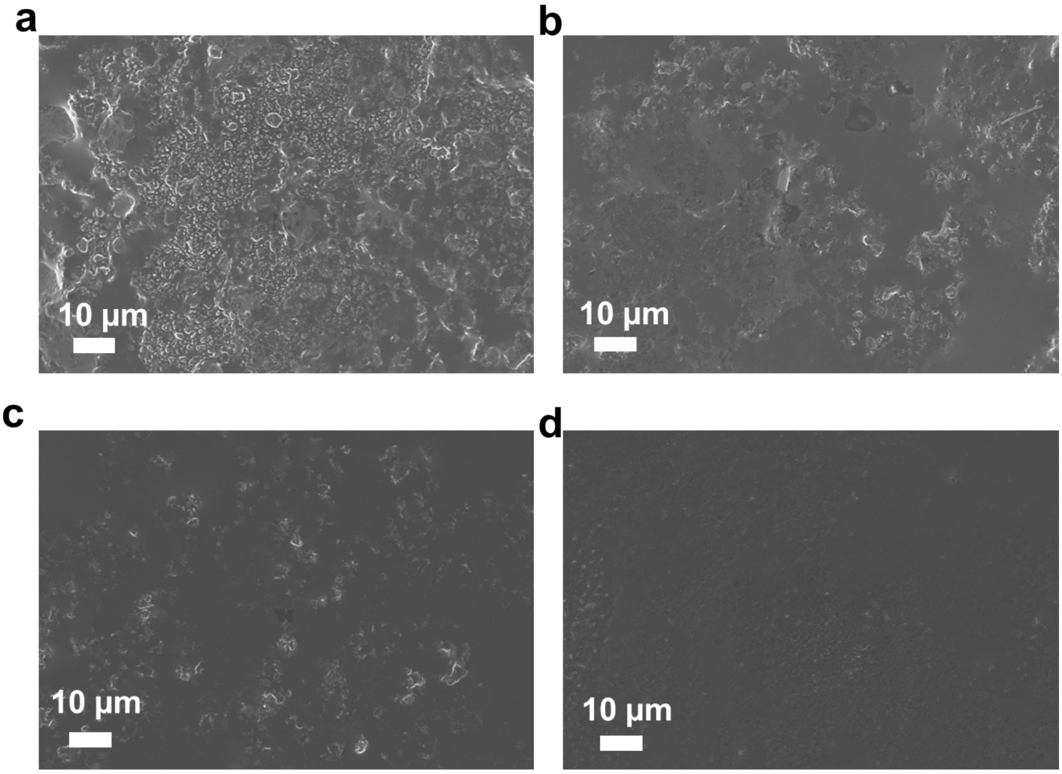

Figure S35. SEM images of SEI morphology on the surface of lithium anode in Li-S cells with different separators after long term cycling, a) PP separator, b) Co@NC-modified separator, c) $Co_{0.85}Se$@NC-modified separator, and d) Co/$Co_{0.85}Se$@NC-modified separator.

Table S1. BET and Pore Volume of the samples.

**Sample**	**Surface area (m^2 g^{-1})**	**Pore volume (cm^3 g^{-1})**
Co/Zn-MOF	1335	0.69
Co@NC	497.87	0.5
$Co_{0.85}Se$@NC	256	0.289
Co/$Co_{0.85}Se$@NC	298.7	0.31

Table S2. Comparison of electrochemical performance of $Co/Co_{0.85}Se$@NC for Li-S batteries with present state-of-the-art catalyst materials.

Electrocatalyst	(Low rate) Cycle number	Reversible capacity (mAh g^{-1})	(High rate) Cycle number	Decay rate (%)	(High rates) Capacity (mAh g^{-1})	Highest area capacity (mAh cm^{-2})	Ref
$Co/Co_{0.85}Se$@NC	(0.5C) 200	932	(2C) 1000	0.042	(3C) 849	10.0	This work
Ni/SiO_2	(0.2C) 100	922	(2C) 300	0.085	(2C) 782	2.7	S[1]
CNT-CoP-Vp	(0.2C) 40	~1000	(2C) 300	0.083	(3C) 738	7.7	S[2]
Co@N-HCMs	(0.2C) 100	~1000	(1C) 500	0.08	(2C) 759	7.3	S[3]
CC@CoSe@HPP	(0.2C) 100	~880	(2C) 1000	0.04	(3C) 754	8.1	S[4]
Co-TiN/C	(0.5C) 200	700			(2C) 752	4.7	S[5]
B/2D MOF-Co	(0.5C) 200	703	(1C) 600	0.07	(2C) 590	7.8	S[6]
$CoSe_2/Co_3O_4$@NC-CNT	(0.2C) 200	881	(2C) 500	0.045	(2C) 778	5.5	S[7]
$W_{0.02}$-Co_3O_4			(1C) 500	0.052	(2C) 741	4.59	S[8]
Co@NC			(1C) 500	0.055	(3C) 741	8.17	S[9]
CoFe PBA	(0.12C) 100	1143	(1.2C) 1000	0.052	(3C) 811	4	S[10]
Co_3O_4-NP/N-rGO	(0.2C) 100	914	(1C) 500	0.058	(3C) 569	~4	S[11]
SeVs-$MoSe_2$	(0.5C) 200	~970	(2C) 400	0.05	(3C) 784.3	6.9	S[12]
RG@CoS			(2C) 420	0.08	(2C) 677	4	S[13]
$Co_{0.9}Zn_{0.1}Te_2$@NC	(0.2C) 300	752	(2C) 1000	0.05	(1C) 1030	8.8	S[14]
EGaSn	(0.1C) 200	~800	(1C) 500		(5C) 526.7	4.5	S[15]
4F-COF	(0.2C) 100	873.1	(2C) 1000		(5C) 660	7.8	S[16]
aCoP@CNTs	(0.2C) 200	814	(1C) 1000	0.049	(2.5C) 795.9		S[17]

References

(1) Chen, C.; Jiang, Q. B.; Xu, H. F.; Zhang, Y. P.; Zhang, B. K.; Zhang, Z. Y.; Lin, Z.; Zhang, S. Q. Ni/SiO2/Graphene-modified separator as a multifunctional polysulfide barrier for advanced lithium-sulfur batteries. *Nano Energy* **2020,** *76*, 105033.

(2) Sun, R.; Bai, Y.; Bai, Z.; Peng, L.; Luo, M.; Qu, M. X.; Gao, Y. C.; Wang, Z. H.; Sun, W.; Sun, K. N. Phosphorus Vacancies as Effective Polysulfide Promoter for High-Energy-Density Lithium-Sulfur Batteries. *Adv. Energy Mater.* **2022,** *12*, 2102739.

(3) Su, L.; Zhang, J. Q.; Chen, Y.; Yang, W.; Wang, J.; Ma, Z. P.; Shao, G. J.; Wang, G. X. Cobalt-embedded hierarchically-porous hollow carbon microspheres as multifunctional confined reactors for high-loading Li-S batteries. *Nano Energy* **2021,** *85*, 105981.

(4) Ye, Z. Q.; Jiang, Y.; Li, L.; Wu, F.; Chen, R. J. A High-Efficiency CoSe Electrocatalyst with Hierarchical Porous Polyhedron Nanoarchitecture for Accelerating Polysulfides Conversion in Li-S Batteries. *Adv. Mater.* **2020,** *32*, 2002168.

(5) Liu, Q. B.; Wu, Y. J.; Li, D.; Peng, Y. Q.; Liu, X. Y.; Li, B. Q.; Huang, J. Q.; Peng, H. J. Dilute Alloying to Implant Activation Centers in Nitride Electrocatalysts for Lithium-Sulfur Batteries. *Adv. Mater.* **2023,** *35*, 2209233.

(6) Li, Y. J.; Lin, S. Y.; Wang, D. D.; Gao, T. T.; Song, J. W.; Zhou, P.; Xu, Z. K.; Yang, Z. H.; Xiao, N.; Guo, S. J. Single Atom Array Mimic on Ultrathin MOF Nanosheets Boosts the Safety and Life of Lithium-Sulfur Batteries. *Adv. Mater.* **2020,** *32*, 1906722.

(7) Chu, R. R.; Nguyen, T. T.; Bai, Y. Q.; Kim, N. H.; Lee, J. H. Uniformly Controlled Treble Boundary Using Enriched Adsorption Sites and Accelerated Catalyst Cathode for Robust Lithium-Sulfur Batteries. *Adv. Energy Mater.* **2022,** *12*, 2102805.

(8) Wang, S. N.; Hu, R. M.; Yuan, D.; Zhang, L.; Wu, C.; Ma, T. Y.; Yan, W.; Wang, R.; Liu, L.; Jiang, X. C.; Liu, H. K.; Dou, S. X.; Dou, Y. H.; Xu, J. T. Single-atomic tungsten-doped Co3O4 nanosheets for enhanced electrochemical kinetics in lithium-sulfur batteries. *Carbon Energy* **2023,** *5*, 329.

(9) Li, Y. J.; Wang, W. Y.; Zhang, B.; Fu, L.; Wan, M. T.; Li, G. C.; Cai, Z.; Tu, S. B.; Duan, X. R.; Seh, Z. W.; Jiang, J. J.; Sun, Y. M. Manipulating Redox Kinetics of Sulfur Species Using Mott-Schottky Electrocatalysts for Advanced Lithium-Sulfur Batteries. *Nano Lett.* **2021,** *21*, 6656-6663.

(10) Chen, Y.; Kang, Y. H.; Yang, H. Y.; Hua, H. M.; Qin, J. X.; Liu, P.; Zhang, Y. Y.; Zhang, Y. J.; Zhao, J. B. Contribution of different metal nodes on stepwise electrocatalysis in lithium-sulfur batteries. *Energy Storage Mater.* **2023,** *54*, 488-497.

(11) Xiao, R. J.; Luo, D.; Wang, J. Y.; Lu, H.; Ma, H.; Akinoglu, E. M.; Jin, M. L.; Wang, X.; Zhang, Y. G.; Chen, Z. W. Oxidation States Regulation of Cobalt Active Sites through Crystal Surface Engineering for Enhanced Polysulfide Conversion in Lithium-Sulfur Batteries. *Adv. Sci.* **2022,** *9*, 2202352.

(12) Wang, M. L.; Sun, Z. T.; Ci, H. N.; Shi, Z. X.; Shen, L.; Wei, C. H.; Ding, Y. F.; Yang, X. Z.; Sun, J. Y. Identifying the Evolution of Selenium-Vacancy-Modulated MoSe2 Precatalyst in Lithium-Sulfur Chemistry. *Angew. Chem. Int. Ed.* **2021,** *60*, 2109291.

(13) Guo, J.; Jiang, H. L.; Li, X. C.; Chu, Z.; Zheng, W. J.; Dai, Y.; Jiang, X. B.; Wu, X. M.; He, G. H. Defective graphene coating-induced exposed interfaces on CoS nanosheets for high redox electrocatalysis in lithium-sulfur batteries. *Energy Storage Mater.* **2021,** *40*, 358-367.

(14) Wang, B.; Wang, L.; Ding, D.; Zhai, Y. J.; Wang, F. B.; Jing, Z. X.; Yang, X. F.; Kong, Y. Y.; Qian, Y. T.; Xu, L. Q. Zinc-Assisted Cobalt Ditelluride

Polyhedra Inducing Lattice Strain to Endow Efficient Adsorption-Catalysis for High-Energy Lithium-Sulfur Batteries. *Adv. Mater.* **2022,** *34*, 2204403.

(15) Qi, Y. Q.; Li, N.; Zhang, K.; Yang, Y.; Ren, Z. Y.; You, J. Y.; Hou, Q.; Shen, C.; Jin, T.; Peng, Z. L.; Xie, K. Y. Dynamic Liquid Metal Catalysts for Boosted Lithium Polysulfides Redox Reaction. *Adv. Mater.* **2022,** *34*, 2204810.

(16) Zhang, K.; Li, X.; Ma, L.; Chen, F. Z.; Chen, Z. X.; Yuan, Y. J.; Zhao, Y. H.; Yang, J. L.; Liu, J.; Xie, K. Y.; Loh, K. P. Fluorinated Covalent Organic Framework-Based Nanofluidic Interface for Robust Lithium-Sulfur Batteries. *Acs Nano* **2023,** *17*, 2901-2911.

(17) Feng, J. A.; Li, J. Y.; Zhang, H. W.; Liu, W. D.; Lin, Z. H.; Wang, T. Y.; Sun, B.; Zhao, X. X.; Wang, F. Y.; Song, J. J. Accelerating redox kinetics by ZIF-67 derived amorphous cobalt phosphide electrocatalyst for high-performance lithium-sulfur batteries. *Energy Materials* **2023,** *3*, 300001.
